# Host cell entry receptors as pharmacological targets in respiratory viral infections: from biology to clinical translation

**DOI:** 10.3389/fcimb.2026.1904701

**Published:** 2026-08-18

**Authors:** Sherif A. El-Kafrawy, Norah A. Othman, Mustafa Zeyadi, Mai M. El-Daly, Esam I. Azhar

**Affiliations:** 1 Special Infectious Agents Unit-BSL3, King Fahd Medical Research Center, King Abdulaziz University, Jeddah, Saudi Arabia; 2 Department of Medical Laboratory Sciences, Faculty of Applied Medical Sciences, King Abdulaziz University, Jeddah, Saudi Arabia; 3 Department of Biochemistry, Faculty of Sciences, King Abdulaziz University, Jeddah, Saudi Arabia

**Keywords:** ACE2, clinical translation, DPP4, host-directed therapy, IgY antibodies, MERS-CoV, pandemic preparedness, RSV

## Abstract

Respiratory viral infections caused by seasonal influenza, respiratory syncytial virus (RSV), and pandemic-potential coronaviruses are responsible for several million hospitalisations and an estimated several hundred thousand deaths each year, a burden underscored by the severe 2024–2025 influenza season and the continued emergence of zoonotic threats such as clade 2.3.4.4b H5N1. This review critically examines the pharmacological basis for targeting host cell-surface receptors as a strategy for the treatment and prophylaxis of respiratory viral infections, evaluates the current evidence for each major receptor axis, and identifies the key translational gaps that must be addressed. Respiratory viruses, including influenza, RSV, SARS-CoV-2, and MERS-CoV, collectively cause millions of hospitalisations annually, and the persistent challenges of antigenic drift, zoonotic emergence, and antiviral resistance highlight the need for mechanistically distinct strategies. Host-directed therapies (HDTs) targeting conserved receptors (ACE2, DPP4, sialic acids, TMPRSS2) exploit genetically stable host factors required for viral entry. Across the receptor axes reviewed here, we appraise more than a dozen candidate agents spanning preclinical development through Phase III evaluation, including TMPRSS2 inhibitors, soluble ACE2 decoys, anti-CD147 and receptor-blocking monoclonal antibodies, the sialidase fusion protein DAS181, and avian IgY preparations. This evidence reveals a consistent gap between robust preclinical activity and as-yet-limited clinical efficacy. We conclude that host receptor targeting is a mechanistically rational but still clinically unproven component of the respiratory antiviral landscape; its value is most plausibly realised in defined niches—prophylaxis, early outpatient treatment, and combination with direct-acting antivirals—and within pandemic preparedness frameworks, provided that the safety, delivery, and trial-design challenges identified here are resolved.

## Introduction

Respiratory viral infections impose an enormous and persistent burden on global health, driving millions of hospitalizations and deaths annually from pathogens as diverse as seasonal influenza, respiratory syncytial virus (RSV), rhinoviruses, and pandemic-potential coronaviruses, including Middle East respiratory syndrome coronavirus (MERS-CoV) and severe acute respiratory syndrome coronavirus 2 (SARS-CoV-2) (Morens and Fauci, 2013; Troeger et al., 2017; Sullivan, 2018). Current countermeasures, vaccines, direct-acting antivirals (DAAs), and passive immunization have each made significant contributions to reducing respiratory viral disease burden, exemplified by the rapid deployment of effective COVID-19 mRNA vaccines, the outpatient benefit of nirmatrelvir-ritonavir and remdesivir, and the marked reductions in infant RSV hospitalization achieved with the monoclonal antibodies nirsevimab and, more recently, clesrovimab. Yet each also faces important limitations: strain specificity that demands continuous reformulation, susceptibility to viral mutational escape, and narrow therapeutic windows that restrict utility in community settings (Hayden and de Jong, 2011; De Clercq and Li, 2016; Zumla et al., 2016). These limitations have intensified interest in an alternative paradigm, host-directed therapy (HDT), which targets the relatively conserved host cell machinery that respiratory viruses depend upon for attachment, entry, and replication, rather than the rapidly evolving viral proteins themselves (Bekerman and Einav, 2015; Kaufmann et al., 2017).

**Figure 1:**
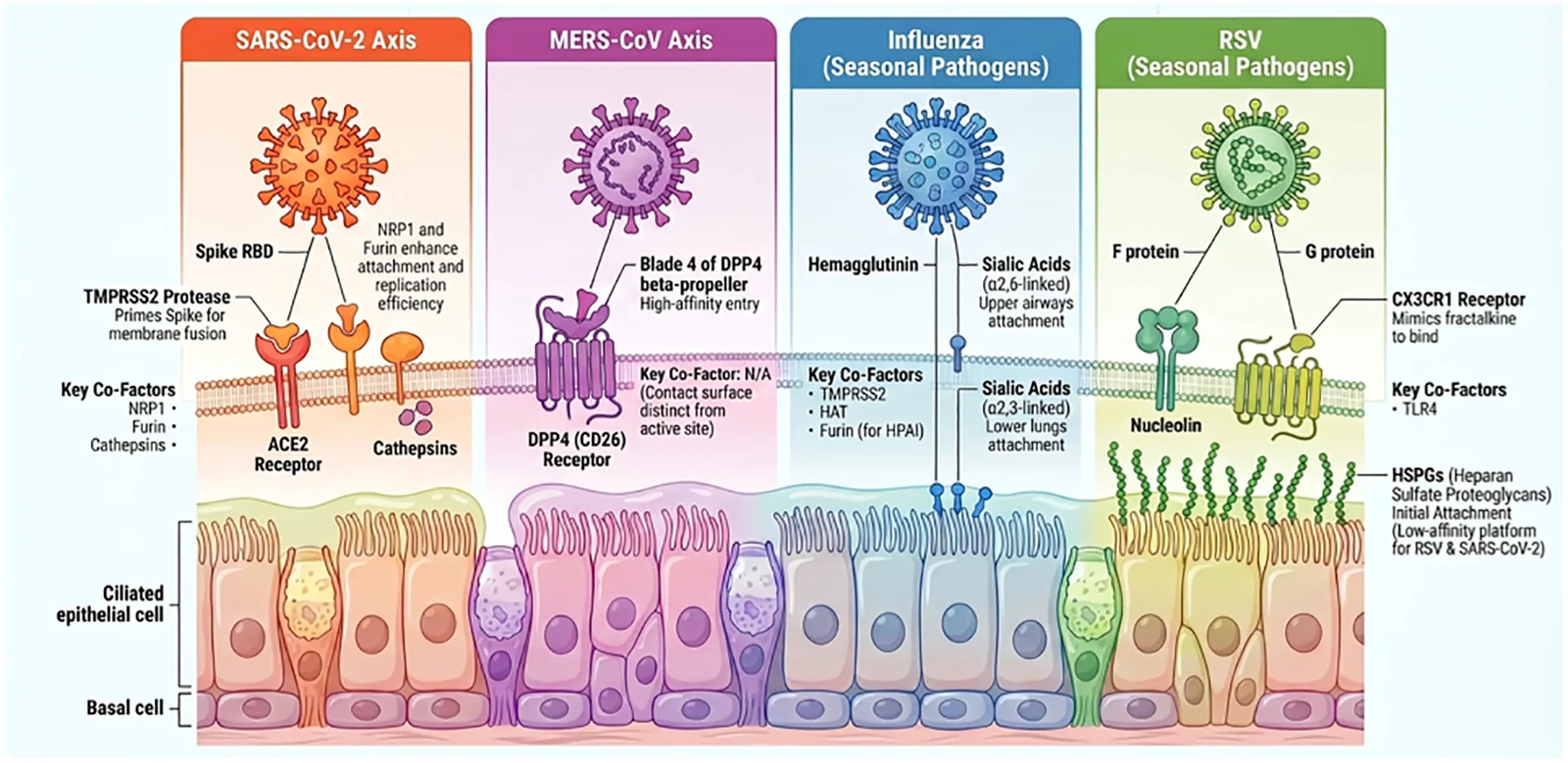
Molecular gateways of major respiratory viruses schematic of host cell entry receptors for four major respiratory pathogens. SARS-CoV-2 engages ACE2 via Spike RBD, primed by TMPRSS2, with NRP1, Furin, and Cathepsins as co-factors. MERS-CoV binds DPP4 blade 4, distinct from the catalytic site. Influenza HA engages sialic acids (α2,6 upper; α2,3 lower airway), requiring TMPRSS2/HAT/Furin for cleavage. RSV uses F protein for fusion and G protein for attachment via Nucleolin, CX3CR1, and HSPGs. Ciliated and basal cells are depicted.

Central to this paradigm are host cell-surface receptors that respiratory viruses exploit for attachment, proteolytic priming, and membrane fusion. These receptors represent therapeutically accessible targets whose genetic stability offers an inherent barrier to viral resistance. (**Figure 1**) (Matrosovich et al., 2009; Raj et al., 2013; Blaas and Fuchs, 2016; Hoffmann et al., 2020). Because these host factors are encoded by the human genome and subject to far slower evolutionary change than viral surface proteins, strategies that block, mimic, or downregulate them offer the theoretical advantage of broad-spectrum activity and a high barrier to viral resistance, while simultaneously raising important considerations regarding the physiological roles of these molecules in cardiovascular, metabolic, immune, and epithelial homeostasis (Zumla et al., 2016).

This review provides a critical pharmacological analysis of host cell entry receptors as drug targets in respiratory viral infections, evaluating the molecular basis of virus–receptor interactions, the therapeutic modalities developed to exploit these interactions, and the clinical evidence supporting their translation from bench to bedside. We evaluate safety considerations, delivery strategies for minimizing systemic toxicity, and the strategic value of host-directed approaches for pandemic preparedness. Together, these considerations position host receptor targeting as a promising and relatively underexplored approach within respiratory antiviral therapy that merits systematic preclinical and clinical development.

### Literature search strategy and scope

This article is a narrative review rather than a systematic review; it aims to synthesize mechanistic, pharmacological, and clinical evidence across multiple receptor axes rather than to answer a single quantitative question. To make the basis for source selection transparent and reproducible, we searched PubMed/MEDLINE, Scopus, and Web of Science from January 2000 to June 2026, supplemented by the ClinicalTrials.gov and EU Clinical Trials registries for trial-stage information and by the reference lists of key articles. Search terms combined receptor and protease identifiers (“ACE2”, “DPP4” OR “CD26”, “TMPRSS2”, “sialic acid”, “neuropilin”, “nucleolin”, “CD147”, “heparan sulfate”) with virus terms (“SARS-CoV-2”, “MERS-CoV”, “influenza”, “respiratory syncytial virus”) and intervention terms (“host-directed”, “receptor blockade”, “soluble decoy”, “monoclonal antibody”, “protease inhibitor”, “antiviral”) using Boolean operators. We prioritised peer-reviewed primary studies, pivotal clinical trials, and authoritative regulatory or public-health sources, and we gave preference to the most recent evidence (2020–2026) where the field was rapidly evolving. Non-English records, conference abstracts without full data, and non-peer-reviewed preprints were generally excluded unless they reported pivotal trial outcomes not yet available elsewhere. Because source selection in a narrative review is necessarily expert-guided rather than protocol-driven, we did not apply a formal risk-of-bias instrument; the implications of this approach, including the potential for selection and publication bias, are addressed explicitly in the Limitations of This Review.

### Burden of respiratory viral infection

Acute respiratory viral infections represent one of the most important causes of lower respiratory tract disease and death worldwide, especially in low- and middle-income countries where access to care and vaccination may be limited (Troeger et al., 2017; Sullivan, 2018). Seasonal and pandemic influenza are estimated to cause approximately 650,000 respiratory deaths annually, while RSV is a leading cause of severe bronchiolitis and pneumonia in infants and young children, and emerging coronaviruses such as MERS-CoV and SARS-CoV-2 have added substantial additional mortality in adults with advanced age or underlying cardiovascular, metabolic, or pulmonary comorbidities (Troeger et al., 2017; Sullivan, 2018; Li, 2020). From a health-systems perspective, recurrent waves of viral respiratory infection drive large numbers of emergency visits and hospital admissions, increase ICU occupancy, and require extensive use of diagnostics, antivirals, oxygen therapy, and vaccination campaigns, all of which translate into high direct medical costs and strain on healthcare resources (Cutler and Summers, 2020). In addition, indirect economic losses arise from school and work absenteeism, long-term disability, and reduced productivity, and a significant proportion of SARS-CoV-2 survivors develop persistent multi-system symptoms, termed post-acute sequelae of COVID-19 or ‘long COVID’, that may persist for months to years, imposing substantial additional burden on individuals, healthcare systems, and economies (Cutler and Summers, 2020; Nalbandian et al., 2021).

### Epidemiological burden and pandemic threats

Respiratory viral infections constitute a persistent epidemiological challenge that transcends geographic and socioeconomic boundaries. Their burden arises not only from endemic circulation but also from episodic emergence of novel pathogens with pandemic potential (Jones et al., 2008; Webster and Govorkova, 2014). Seasonal influenza viruses infect 5-15% of the global population each year, leading to an estimated 3–5 million severe cases and up to 650,000 deaths annually (Sullivan, 2018). However, antigenic drift within circulating influenza A and B strains necessitates annual vaccine updates and at times reduces vaccine efficacy, particularly in older or immunocompromised individuals (Belongia et al., 2016; Skowronski et al., 2019). Periodic antigenic shift events, when reassortment generates a novel hemagglutinin or neuraminidase subtype, can ignite pandemics, as witnessed in the devastating 1918 H1N1 pandemic and later H2N2, H3N2, and H1N1pdm09 events (Taubenberger and Morens, 2010).

Beyond influenza, zoonotic coronaviruses have emerged as major respiratory threats in the twenty-first century. SARS-CoV in 2002–2003 caused roughly 8,000 confirmed infections with nearly 10% mortality, while MERS-CoV since 2012 has maintained an even higher case-fatality rate exceeding 30%, largely linked to dromedary camel exposure in the Middle East (Zaki et al., 2012; Memish et al., 2020). The most consequential emergence, SARS-CoV-2, evolved into an unprecedented global pandemic responsible for tens of millions of excess deaths and severe socioeconomic disruption (Cutler and Summers, 2020; Li, 2020). Viral evolution through mutations in the spike protein has generated multiple variants with enhanced transmissibility, immune escape, or altered pathogenic potential, demonstrating the continuous dynamic between viral adaptation and human immunity (Kemp et al., 2021).

Environmental and societal factors amplify these threats. Rapid urbanization, international travel, and intensive animal husbandry collectively amplify the risk of cross-species viral transmission and accelerate global pandemic spread (Plowright et al., 2017). Climate change, deforestation, and habitat encroachment may further reshape virus-host ecology, expanding the geographic range of zoonotic reservoir hosts and extending transmission seasons for respiratory viruses (Carlson et al., 2022). These converging pressures highlight the necessity for integrated “One Health” surveillance that couples human, animal, and environmental monitoring to anticipate and mitigate the next pandemic threat.

### Limitations of virus-targeted therapeutics (resistance, specificity)

Therapeutic strategies that directly target viral proteins have historically achieved only partial success and exhibit several critical limitations related to specificity and resistance. Many approved antivirals, such as oseltamivir and zanamivir for influenza, or remdesivir for SARS-CoV-2, act on essential viral enzymes like neuraminidase or RNA-dependent RNA polymerase (Hayden and de Jong, 2011; Dunning et al., 2014). However, these agents are typically virus-specific and vulnerable to resistance (e.g., H275Y neuraminidase substitution in H1N1 influenza conferred widespread oseltamivir resistance without loss of viral fitness (Bloom et al., 2010), while SARS-CoV-2 variants with altered polymerase or exonuclease activity have shown decreased remdesivir susceptibility (Pauly et al., 2017). More recently, serial passage of SARS-CoV-2 under the 3CLpro inhibitor nirmatrelvir has selected resistance substitutions in the main protease (e.g., L50F, E166A/V, and L167F), and related mutations have been documented in immunocompromised patients receiving prolonged therapy, confirming that even the newest oral DAAs are not immune to the resistance problem that motivates host-directed alternatives (Jochmans et al., 2023).

Equally problematic is the narrow therapeutic window, many direct antivirals are effective only when administered within the first 24–48 hours of symptom onset, before peak viral replication (Dunning et al., 2014). Real-world delays in diagnosis, limited access to early testing, or asymptomatic incubation severely constrain their utility in community settings. The development pipeline faces additional challenges: preclinical screening of compounds requires high-biosafety facilities, viral diversity complicates selection of conserved drug targets, and the emergence of immune-evasive variants demands continual reformulation (Zumla et al., 2016).

Moreover, monotherapy approaches often fail to account for host heterogeneity and immune-mediated pathology. Severe viral pneumonias result not only from direct cytopathic effects but also from dysregulated host inflammation, suggesting that therapies targeting only viral replication may be insufficient for preventing clinical deterioration (Merad and Martin, 2020). Consequently, current research emphasizes host-directed therapies, such as modulators of interferon signaling, viral receptor blocking agents, or metabolic pathway regulators, that could provide broader antiviral coverage and higher barriers to resistance. Combination regimens that integrate such host-targeted strategies with direct antiviral or immunomodulatory agents hold promise for both improved efficacy and pandemic preparedness (Zumla et al., 2016; Dyall et al., 2017). Recent medicinal-chemistry work illustrates how this integration can be achieved within a single molecule: rationally designed dual PB2/JAK2 inhibitors for influenza A combine direct antiviral activity against the viral polymerase cap-binding subunit with host-directed suppression of the JAK-STAT signaling that drives virus-induced hyperinflammation, achieving a balanced antiviral and immunomodulatory effect in preclinical models (Rong et al., 2026; Yang et al., 2026). Although such dual-target agents lie outside the entry-receptor focus of this review, they exemplify the broader principle that pairing direct-acting and host-directed mechanisms can simultaneously improve efficacy and raise the barrier to resistance.

### Vaccines as preventive human-targeted interventions

Vaccination remains the most effective strategy for reducing the population-level burden of many viral infections by inducing durable adaptive immune responses against viral antigens (Plotkin, 2014; Krammer, 2020). For respiratory viruses, platforms used include inactivated and live-attenuated vaccines (e.g., seasonal influenza), protein subunit vaccines, viral-vector vaccines, and nucleic-acid vaccines, the latter having been rapidly deployed and optimized during the COVID-19 pandemic (Krammer, 2020; Polack et al., 2020; Baden et al., 2021). These vaccines primarily target viral surface proteins, particularly the coronavirus spike glycoprotein, to elicit neutralizing antibodies that prevent receptor engagement and cell entry (Krammer, 2020). However, antigenic drift and shift in influenza and the emergence of SARS-CoV-2 variants with altered receptor-binding domains illustrate the challenge of maintaining vaccine effectiveness and motivate the design of broadly protective or pan-sarbecovirus vaccines (Burton and Walker, 2020; Krammer, 2020).

### Passive immunization and antibody-based therapies

Passive immunization through exogenous immunoglobulins provides immediate protection during the window between pathogen emergence and vaccine availability (Casadevall and Scharff, 1995; Graham and Ambrosino, 2015). These interventions encompass conventional polyclonal immunoglobulin and hyperimmune sera derived from pooled or specifically immunized donors, highly specific monoclonal antibodies (mAbs), and alternative formats such as egg-derived IgY, each characterized by distinct pharmacokinetic profiles and effector functions (Rahman et al., 2013; Graham and Ambrosino, 2015). Neutralizing antibodies act principally by binding viral surface proteins, such as the receptor-binding domain of coronavirus spike, and thereby blocking interactions with host receptors like DPP4 or ACE2, or constraining the conformational changes required for membrane fusion, while Fc regions additionally mediate antibody-dependent cellular cytotoxicity, antibody-dependent cellular phagocytosis, and complement activation (Saphire et al., 2018; Tay et al., 2020). In clinical practice, mAbs have been successfully developed for the prevention of severe RSV disease in high-risk infants and for early treatment or prophylaxis of COVID-19 in vulnerable adults, with several products engineered to extend serum half-life and optimize Fc-mediated effector functions, although their efficacy can be compromised by ongoing viral antigenic evolution (Graham and Ambrosino, 2015; Corti et al., 2021). IgY-based preparations from immunized hens offer scalable, cost-effective passive immunisation with favourable mucosal delivery properties (Nguyen et al., 2010; Rahman et al., 2013). Taken together, these modalities position passive antibody therapy as a crucial adjunct to vaccination and direct-acting antivirals, particularly for individuals at high risk of severe disease or in need of rapid protection during outbreaks (Casadevall and Scharff, 1995; Corti et al., 2021).

### Direct-acting antiviral agents (chemical antivirals)

Direct-acting antiviral agents (DAAs) target viral enzymes essential for replication and virion release (De Clercq and Li, 2016). Mechanistically, major classes include polymerase inhibitors, which block viral RNA- or DNA-dependent polymerases; protease inhibitors, which prevent cleavage of viral polyproteins into functional units; and entry or fusion inhibitors, which interfere with the conformational changes or receptor interactions required for membrane fusion and cell entry (De Clercq and Li, 2016). In the context of respiratory viruses, neuraminidase inhibitors (oseltamivir, zanamivir) and polymerase/protease inhibitors (remdesivir, nirmatrelvir) provide clinical benefit when given early but face limitations including narrow spectrum, resistance emergence, and pathogen-specificity that motivate interest in targeting conserved host factors (Hayden and de Jong, 2011; McKimm‐Breschkin, 2012). For SARS-CoV-2, DAAs including remdesivir (RdRp inhibitor) and nirmatrelvir (3CLpro inhibitor) provide clinical benefit when given early but face resistance risks (De Clercq and Li, 2016; Singh et al., 2021). When administered early to high-risk outpatients, these agents can significantly reduce viral load and lower the risk of hospitalization or progression to severe COVID-19, emphasizing the importance of timing for DAAs in acute respiratory infections (Singh et al., 2021). Although DAAs are usually directed against viral proteins, some agents indirectly modulate host pathways required for replication, and resistance may emerge through viral mutations, especially under suboptimal dosing, incomplete adherence, or widespread use (McKimm‐Breschkin, 2012; De Clercq and Li, 2016). Combination regimens and the development of broad-spectrum antivirals that target conserved viral elements or shared replication mechanisms are strategies under investigation to mitigate resistance and improve effectiveness in severe respiratory infections (Zumla et al., 2016).

## Rationale for host-directed therapies

**Figure 2:**
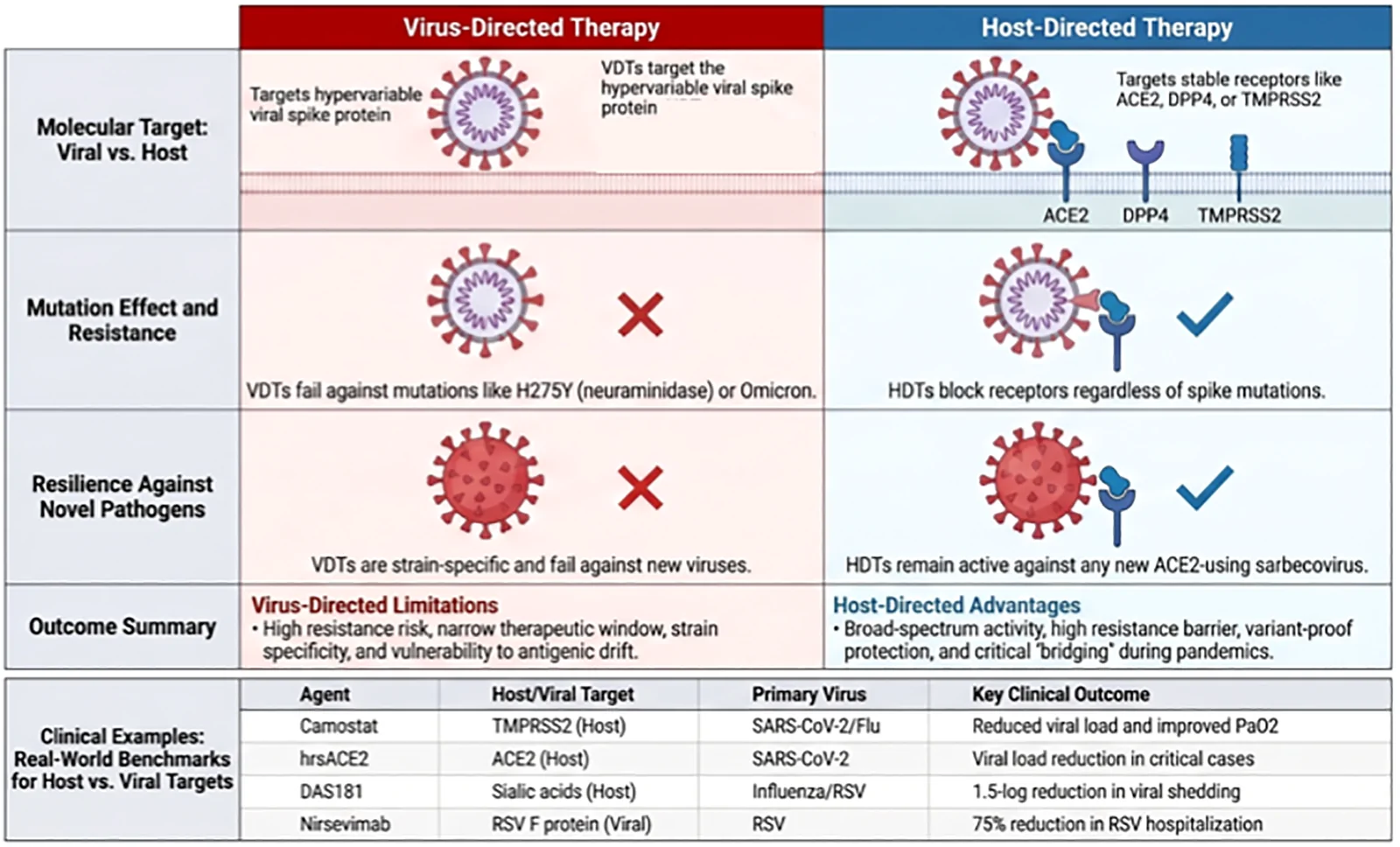
Host-directed vs. virus-directed therapy comparative schematic illustrating HDTs vs. VDTs across four dimensions: molecular target stability, resistance profiles, resilience against novel pathogens, and outcome summary. HDTs targeting shared host dependencies (e.g., ACE2 for sarbecoviruses) retain broad-spectrum activity against novel pathogens using the same entry pathway. Outcome Summary (Row 4): VDTs carry higher resistance risk and strain specificity but benefit from extensive clinical validation, established regulatory pathways, and proven efficacy in licensed products (e.g., oseltamivir, nirmatrelvir); HDTs offer potentially broader-spectrum coverage and a higher barrier to resistance, but face challenges including limited clinical efficacy data, on-target safety concerns from blockade of physiologically essential host proteins, delivery optimisation requirements, and a less mature regulatory framework. Clinical Benchmarks (bottom): Four representative agents illustrate the current clinical development landscape: camostat mesylate (TMPRSS2 inhibitor; modest antiviral effects in some Phase II cohorts, though results have been inconsistent); hrsACE2 (soluble ACE2 decoy; viral load reduction observed in a compassionate-use case report); DAS181 (sialidase; viral shedding reduction in Phase II trials); and nirsevimab† (RSV F protein-targeting mAb; 75% reduction in RSV-associated hospitalisation in the Phase III MELODY trial). †Nirsevimab targets the viral RSV F protein rather than a host receptor, and is included as a licensed benchmark comparator to contextualise the clinical maturity of host-directed approaches relative to established virus-directed prophylaxis. VDT, virus-directed therapy; HDT, host-directed therapy; ACE2, angiotensin-converting enzyme 2; DPP4, dipeptidyl peptidase-4; TMPRSS2, transmembrane serine protease 2; PaO_2_, partial pressure of arterial oxygen; RSV, respiratory syncytial virus.

The pharmacological rationale for targeting host entry factors rests on two principles. First, host receptors are encoded by the human genome and constrained by essential physiological functions, making them refractory to the rapid mutational escape that limits virus-directed agents. Second, the convergent dependence of phylogenetically diverse viruses on a small number of conserved host factors (ACE2, DPP4, sialic acids, TMPRSS2) creates the possibility of broad-spectrum pharmacological intervention. These principles and their therapeutic implications are illustrated in **Figure 2**.

### Human receptors as viral entry targets and the case for host-directed intervention

For respiratory coronaviruses, the best-defined host entry receptors are dipeptidyl peptidase-4 (DPP4/CD26) for MERS-CoV and angiotensin-converting enzyme 2 (ACE2) for SARS-CoV and SARS-CoV-2 (Raj et al., 2013; Hoffmann et al., 2020). Both receptors can be blocked or mimicked pharmacologically. Sialic acid residues can be enzymatically removed from airway surfaces, while TMPRSS2 represents the most broadly shared host dependency, amenable to pan-viral protease inhibitors regardless of annual vaccine mismatch or emergent pandemic strain (Wang et al., 2013; Monteil et al., 2020).

This evolutionary invariance underpins the HDT paradigm. To evade a host-directed agent, a virus would need to switch to an alternative receptor, a fundamental tropism alteration unlikely under therapeutic selective pressure. This positions HDTs as potentially critical first-line responses in the early weeks of an outbreak, before pathogen-specific countermeasures become available.

### Therapeutic approaches: receptor blockade, decoys, and chemical inhibitors

Three mechanistic approaches to host receptor targeting are central to this review. The first is direct receptor blockade using monoclonal or polyclonal antibodies that bind epitopes overlapping the viral contact surface on DPP4 or ACE2, sterically hindering spike-receptor engagement and the conformational changes required for membrane fusion (Haga et al., 2008; Raj et al., 2013; Monteil et al., 2020; Walls et al., 2020). Such receptor-targeted antibodies retain activity across viral variants provided the host receptor epitope remains conserved, a potential advantage over antibodies directed solely at the hypervariable regions of the viral spike protein.

Recombinant soluble receptor decoys (e.g., sACE2-Fc, sDPP4) intercept virions extracellularly before cell contact, acting as competitive traps with engineerable pharmacokinetics.block both receptor-binding and proteolytic activation steps required for productive viral entry (Monteil et al., 2020).

The third approach involves small-molecule modulators that downregulate receptor surface expression, alter receptor conformation, or inhibit associated co-receptors and host proteases such as TMPRSS2 and cathepsin L (Simmons et al., 2013; Hoffmann et al., 2020). In parallel, avian IgY polyclonal antibody preparations target multiple epitopes simultaneously, imposing a higher genetic barrier to viral escape than single-epitope monoclonal antibodies, while offering logistical advantages, including low-cost production and suitability for topical mucosal delivery, particularly relevant in resource-limited and outbreak-response settings (Nguyen et al., 2010; Rahman et al., 2013).

While Kumar et al. (2020) recently published a comprehensive review on host-directed antiviral therapy emphasizing cellular mechanisms and target discovery, the present review provides a focused, mechanistically detailed analysis of the four major host cell entry receptors exploited by respiratory viruses (ACE2, DPP4, TMPRSS2, and sialic acid), with emphasis on structural pharmacology of virus-receptor interactions, the therapeutic modalities developed to exploit each receptor axis, and a critical appraisal of Phase I–III clinical trial outcomes that have revealed significant gaps between preclinical promise and clinical translation.

Together, these approaches create multiple independent layers of entry blockade. The remainder of this review evaluates the evidence, examines clinical development, and identifies gaps that must be addressed for clinical translation of combination antiviral regimens for both treatment and pandemic preparedness.

## Major respiratory viruses and their host receptors

The principal virus-receptor axes reviewed in this section are summarized in **Figure 1**.

### DPP4: primary entry receptor for MERS-CoV

#### Molecular biology and structure of the DPP4-spike interface

Dipeptidyl peptidase 4 (DPP4; also designated CD26) serves as the obligate entry receptor for Middle East respiratory syndrome coronavirus (MERS-CoV), a betacoronavirus with a case-fatality rate exceeding 30% that has caused over 2,600 confirmed human infections since its identification in 2012, with the highest burden concentrated in Saudi Arabia and the broader Arabian Peninsula (Zaki et al., 2012; Memish et al., 2020). DPP4 is a type II transmembrane serine exopeptidase existing as a homodimer, with each monomer comprising a catalytic domain and an eight-bladed beta-propeller domain (Raj et al., 2013). Critically, the MERS-CoV receptor-binding domain (RBD) of the spike protein engages DPP4 through a surface that is entirely distinct from the enzyme’s catalytic active site, explaining why DPP4 inhibitors currently licensed as antidiabetic agents (sitagliptin, saxagliptin, vildagliptin), which occupy the enzyme’s substrate-binding pocket, do not competitively block MERS-CoV entry (Lu et al., 2013; Wang et al., 2013).

High-resolution crystallographic studies have precisely defined the DPP4-MERS-CoV RBD interface (Lu et al., 2013; Wang et al., 2013). The MERS-CoV RBD (residues 367-606) contacts DPP4 blade 4 through an extensive interface dominated by hydrophobic and hydrogen-bond contacts (Wang et al., 2013). This binding interface is remarkably different from the ACE2-SARS-CoV-2 spike interface in both structural geometry and the amino acids involved, yet results in a comparable binding affinity (Kd ~16 nM for DPP4-MERS-CoV RBD versus Kd ~15–30 nM for ACE2-SARS-CoV-2 RBD), establishing DPP4 as a high-affinity, high-priority receptor target for therapeutic intervention (Lu et al., 2013; Walls et al., 2020). Point mutations at the interface, particularly DPP4 K267A or R317A, abolish MERS-CoV pseudovirus entry without affecting enzyme activity, confirming that the viral attachment surface is structurally separable from the catalytic function of DPP4 and validating the therapeutic concept of selectively blocking the viral contact surface while preserving enzymatic activity (Wang et al., 2013).

#### Tissue expression, tropism, and pathogenesis

DPP4 is expressed on airway epithelial cells, renal tubule cells, hepatocytes, enterocytes, and immune cells (T cells, NK cells, macrophages) (Raj et al., 2013; Meyerholz et al., 2016). This broad distribution explains the multi-organ pathology of severe MERS-CoV infection (Assiri et al., 2013; Arabi et al., 2017). The expression of DPP4 on T lymphocytes provides a mechanistic explanation for the profound lymphopenia observed in severe MERS-CoV infection, as direct T cell infection or receptor-mediated immune dysregulation may deplete critical antiviral effector populations (Zhou et al., 2013; Chu et al., 2015). Furthermore, DPP4 expression has been shown to be upregulated in the metabolic syndrome, hyperglycemia, obesity, and insulin resistance, which may partly explain the disproportionate severity of MERS-CoV in diabetic patients (Assiri et al., 2013; Meyerholz et al., 2016).

Species-specific variation in DPP4 sequence at the viral contact residues determines host susceptibility to MERS-CoV. Dromedary camels (Camelus dromedarius), the principal zoonotic reservoir, possess DPP4 with identical contact residues to human DPP4 at the critical binding interface, explaining efficient camel-to-human transmission (Müller et al., 2012; Reusken et al., 2013). By contrast, murine DPP4 differs at multiple key contact residues (positions 288 and 330 in mice versus humans), rendering wild-type mice resistant to MERS-CoV infection, a barrier that has necessitated the development of transgenic hDPP4-knock-in mouse models and human DPP4-expressing adeno-associated virus (AAV-hDPP4) models for preclinical evaluation of therapeutic candidates (Zhao et al., 2014). This species barrier has been a significant impediment to MERS-CoV therapeutic development and must be considered when interpreting preclinical efficacy data for DPP4-targeting agents.

### Therapeutic strategies targeting DPP4

#### Receptor-blocking monoclonal antibodies

Anti-DPP4 monoclonal antibodies that bind epitopes overlapping the MERS-CoV spike contact surface represent the most extensively characterized class of DPP4-directed therapeutics. Several antibodies have been identified and characterized, including MERS-4 and MERS-27, which target overlapping epitopes on the blade 4–5 interface of DPP4 and potently inhibit MERS-CoV pseudovirus entry (IC_50_ 0.005-0.05 μg/mL) without affecting DPP4 enzymatic activity toward its physiological substrates (Raj et al., 2014). The structural rationale for this selectivity has been confirmed crystallographically: antibody epitopes on the convex face of the beta-propeller domain are spatially remote from the substrate-binding pocket located at the apex of the catalytic domain, allowing complete steric blockade of viral docking while leaving incretin cleavage intact (Wang et al., 2013; Raj et al., 2014). In humanized DPP4 mouse models and non-human primates expressing human DPP4, prophylactic administration of anti-DPP4 antibodies reduced lung viral titers by 3–4 logs and prevented ARDS-like pathology, establishing *in vivo* proof of concept for this approach (Zhao et al., 2014).

**Figure 3:**
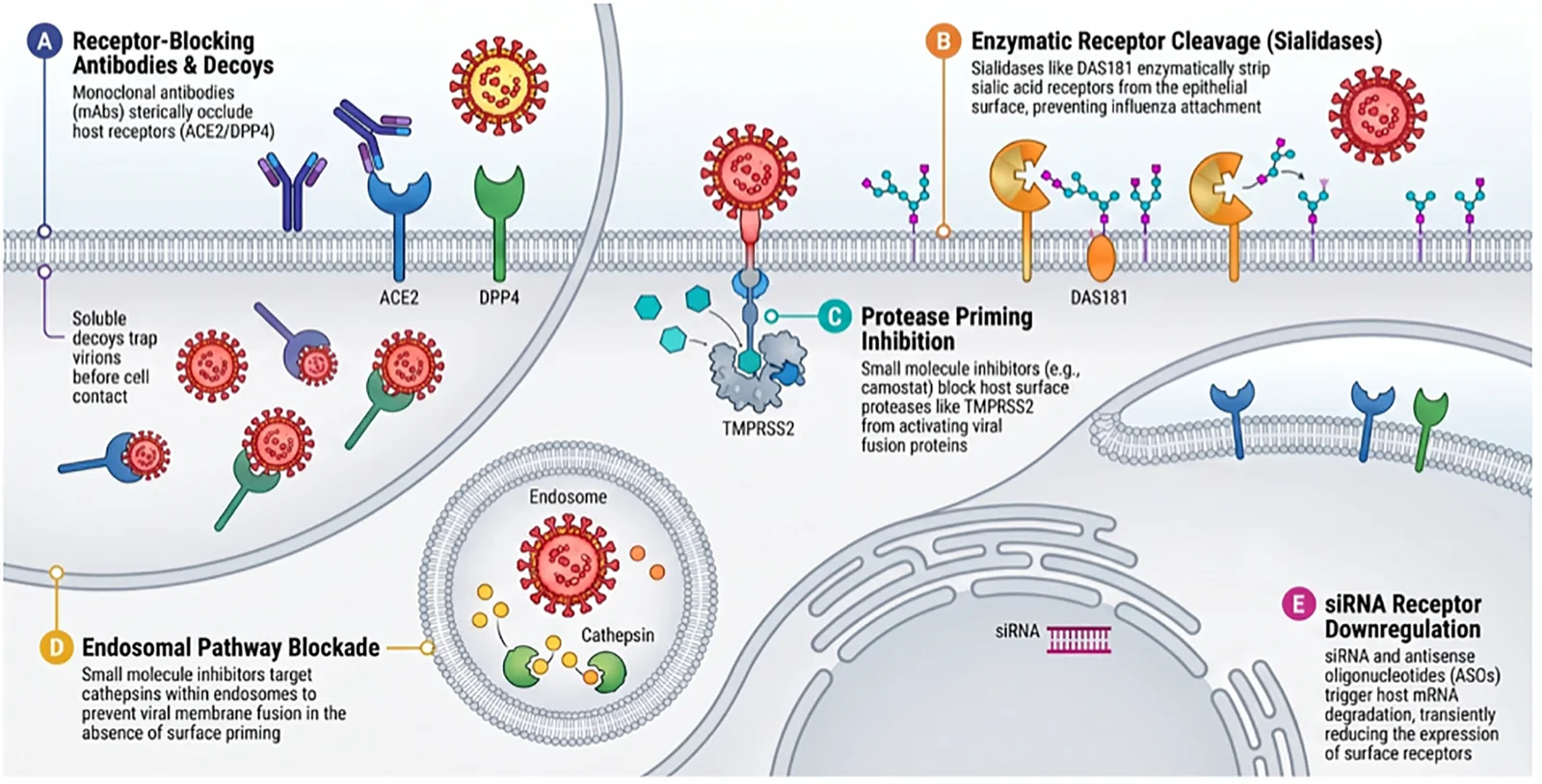
Mechanisms of action for host-directed antiviral modalities. Five strategies targeting host factors: **(A)** Receptor-Blocking Antibodies/Decoys occlude host receptors or intercept virions. **(B)** Sialidases (DAS181) cleave sialic acid from airway epithelium. **(C)** Protease Inhibition (camostat/nafamostat) blocks TMPRSS2-mediated activation. **(D)** Cathepsin inhibitors block endosomal entry. **(E)** siRNA/ASO downregulate receptor expression, limiting viral entry.

Cross-reactive antibodies targeting DPP4 have shown the potential to block not only MERS-CoV but also related bat betacoronavirus strains that use DPP4 orthologs as entry receptors, supporting the development of pan-DPP4-blocking antibodies as broad-spectrum pre-pandemic countermeasures against the MERS-CoV clade of betacoronaviruses (Zhao et al., 2014). Bispecific antibody formats pairing anti-DPP4 blockade with anti-MERS-CoV spike neutralization have been proposed as a strategy to achieve dual inhibition, preventing receptor engagement and neutralizing free virions simultaneously, analogous to combination mAb strategies used for SARS-CoV-2 (Corti et al., 2021). IgY antibody preparations raised against the MERS-CoV RBD-DPP4 interface have been explored for intranasal delivery, capitalizing on the same mucosal delivery advantages demonstrated for other respiratory pathogens (Abbas et al., 2019; El-Kafrawy et al., 2021). Receptor-blocking mAbs sterically occlude viral RBD-host interfaces, while soluble decoys intercept virions extracellularly before cell contact (**Figure 3A**).

#### Soluble DPP4 decoys

Recombinant soluble DPP4 (sDPP4) functions as a viral decoy, competing with membrane-bound DPP4 for spike binding (Lu et al., 2013; Raj et al., 2013). Monomeric sDPP4 binds MERS-CoV RBD with a Kd of approximately 16 nM, comparable in affinity to cell-surface DPP4; dimeric Fc-fusion formats (sDPP4-Fc) increase avidity 10-50-fold through bivalent spike engagement, achieving IC_50_ values of 0.1-1 μg/mL in pseudovirus neutralization assays (Lu et al., 2013). Engineered high-affinity sDPP4 variants carrying interface-optimizing mutations (e.g., L294W, A291P) further enhance binding affinity 5-10-fold, translating to proportionally improved neutralization potency against authentic MERS-CoV in Vero and Calu-3 cells (Richard and Fouchier, 2015). Critically, sDPP4 decoys retain full enzymatic activity toward GLP-1 and other physiological substrates, an important safety feature given the metabolic consequences of DPP4 suppression in diabetic patients, the very population at highest risk for severe MERS-CoV disease (Meyerholz et al., 2016). Intranasal administration of sDPP4-Fc in hDPP4-expressing mice achieved airway concentrations exceeding 10 μg/mL at 4 hours post-dose and conferred 80-90% protection against lethal MERS-CoV challenge, supporting localized respiratory delivery as the preferred route (Zhao et al., 2014).

#### Small molecule approaches and DPP4 inhibitor repurposing

Novel small molecules designed to allosterically stabilize the DPP4 homodimer in a conformation that sterically excludes MERS-CoV RBD docking, without occupying the catalytic pocket, represent a more rational pharmacological approach (Raj et al., 2013; Wang et al., 2013). Computational virtual screening of the DPP4 viral contact surface has identified candidate compounds that dock at the blade 4 interface with predicted IC_50_ values in the micromolar range, though *in vitro* validation of lead compounds remains in early stages (Mustafa et al., 2018). This allosteric approach, if validated, could provide an orally bioavailable, small-molecule DPP4 blocker that combines antiviral entry inhibition with the metabolic benefits of incretin pathway modulation, a particularly attractive profile for the diabetic MERS-CoV patient population.

#### Physiological safety considerations for DPP4 targeting

DPP4 serves multiple physiological functions including regulation of incretin hormones (GLP-1, GLP-2), chemokines (SDF-1), glucose homeostasis, and immune cell trafficking (Drucker, 2007). Complete DPP4 knockout in rodents produces mild phenotypic abnormalities including enhanced insulin sensitivity, slightly elevated blood pressure, and immune dysregulation, suggesting that partial or transient DPP4 blockade at the viral contact surface, particularly when delivered locally to the respiratory tract, is unlikely to produce clinically significant metabolic or immune perturbations (Drucker, 2007; Raj et al., 2013). This favorable safety profile compared to ACE2 or TMPRSS2 targeting, where cardiovascular and epithelial biology consequences are more significant, makes DPP4 a particularly tractable host receptor target from a therapeutic safety standpoint (Raj et al., 2013; Meyerholz et al., 2016). The extensive clinical safety database accumulated from >10 years of licensed gliptin use in hundreds of millions of diabetic patients worldwide also provides strong indirect reassurance about the tolerability of DPP4 modulation, although the viral-contact-surface-blocking strategy differs mechanistically from catalytic site inhibition (Drucker, 2020).

A separate body of clinical evidence concerns the antidiabetic gliptins themselves. Because these agents do not occupy the MERS-CoV spike-binding surface of DPP4, any benefit in coronavirus infection would be expected to arise from immunomodulatory rather than entry-blocking mechanisms, and the evidence here must be read at the level of observational clinical data rather than receptor pharmacology. Several retrospective cohorts and a case–control study of hospitalized patients with type 2 diabetes and COVID-19 reported lower mortality in DPP4-inhibitor users, for example, a multicenter Saudi cohort in which most exposed patients received sitagliptin, and an Italian study reporting reduced mortality and ventilation with sitagliptin started at admission. A meta-analysis of predominantly retrospective studies estimated a pooled mortality odds ratio of approximately 0.75. However, other cohorts found no benefit or even worse outcomes, and all are subject to confounding by indication and diabetes severity. These findings should therefore be regarded as hypothesis-generating signals about immunomodulation rather than as evidence that DPP4-directed agents block MERS-CoV or SARS-CoV-2 entry; they reinforce, rather than substitute for, the need for prospective trials of receptor-contact-surface-blocking strategies (Drucker, 2020; Soens et al., 2025).

#### DPP4 in the context of pandemic preparedness

MERS-CoV’s persistence as an endemic zoonosis in the Middle East, its high fatality rate, its capacity for healthcare-associated amplification, and its potential for pandemic emergence if transmissibility-enhancing mutations accumulate in the spike protein make DPP4-targeting strategies a high-priority component of respiratory coronavirus pandemic preparedness planning (Zumla et al., 2016; Dyall et al., 2017). Unlike SARS-CoV-2, which achieved high-efficiency human-to-human transmission largely through ACE2 affinity optimization, MERS-CoV has not yet acquired the transmissibility characteristics needed for sustained pandemic spread, but this barrier is not absolute, and surveillance data document ongoing evolution in circulating dromedary MERS-CoV strains (Müller et al., 2012; Memish et al., 2020). Pre-positioning stockpiles of validated anti-DPP4 agents represents a rational preparedness strategy (Dyall et al., 2017). The continued circulation of MERS-CoV on the Arabian Peninsula, where the great majority of human cases have been reported, underscores the clinical urgency of advancing DPP4-directed therapeutics through rigorous clinical evaluation.

### SARS-CoV-2 and coronaviruses

SARS-CoV-2, alongside other betacoronaviruses, exemplifies how receptor adaptation drives zoonotic emergence and human-to-human spread within the Coronaviridae family (Zhou et al., 2013; Andersen et al., 2020). Seasonal coronaviruses like HCoV-OC43 and HCoV-HKU1 cause mild upper respiratory illness through sialic acid engagement, whereas highly pathogenic SARS-CoV (2003) and SARS-CoV-2 (2019) evolved spike-mediated binding to ACE2, a metalloprotease enriched in lung alveolar cells, enabling lower respiratory tropism and severe pneumonia (Lan et al., 2020). MERS-CoV diverges by using DPP4 on nonciliated bronchial epithelia, contributing to its focal Middle Eastern epidemiology and high fatality (Raj et al., 2013). Alphacoronaviruses HCoV-229E and NL63 use APN and ACE2, respectively, highlighting receptor promiscuity that facilitates bat-to-human spillover via intermediate hosts like civets or camels (Graham and Baric, 2010). Post-binding, SARS-CoV-2 spike requires host proteases, TMPRSS2 for surface cleavage or cathepsin B/L for endosomal activation, coupling receptor engagement to membrane fusion and cytosolic entry, a conserved mechanism vulnerable to broad-spectrum inhibitors like camostat (Hoffmann et al., 2020). The acquisition of a polybasic furin cleavage site in SARS-CoV-2 spike further enhances multicycle replication efficiency in human airways, contributing to its exceptional pandemic transmissibility.

#### ACE2: primary receptor for SARS-CoV-2 and SARS-CoV

Angiotensin-converting enzyme 2 (ACE2), a zinc metallopeptidase expressed on respiratory epithelial cells, serves as the primary entry receptor for SARS-CoV and SARS-CoV-2, forming a high-affinity interface with the viral spike receptor-binding domain (RBD) (Li et al., 2005; Shang et al., 2020). Expressed constitutively on type II pneumocytes, endothelial cells, and enterocytes, ACE2 modulates the renin-angiotensin system and exerts protective effects against lung injury, but its downregulation during SARS-CoV-2 infection may exacerbate coagulopathy and fibrosis through angiotensin II accumulation (Glowacka et al., 2011). SARS-CoV-2 exhibits 10- to 20-fold higher ACE2-binding affinity than SARS-CoV, attributable to optimized contacts within the receptor-binding motif (including key substitutions such as N501Y) that stabilize the spike-ACE2 interface, correlating with superior nasal transmissibility and asymptomatic shedding (Wrapp et al., 2020). Accessory roles for neuropilin-1 (NRP1) and heparan sulfates as co-factors enhance ACE2 engagement, particularly for emerging variants, while basolateral expression in polarized epithelia facilitates systemic dissemination (Cantuti-Castelvetri et al., 2020). Therapeutically, soluble ACE2 decoys or anti-ACE2 antibodies offer receptor trap strategies, preserving endogenous ACE2 while blocking viral access, with preclinical efficacy against pseudotyped sarbecoviruses (Lei et al., 2020). This receptor conservation across sarbecoviruses positions ACE2 blockade as a prototype for HDTs targeting pivotal host dependencies.

The continued evolution of SARS-CoV-2 through the XBB, EG.5, and BA.2.86/JN.1 lineages illustrates why a host-receptor strategy is mechanistically attractive even as the virus diversifies. Across these Omicron descendants, the spike has been under dual selection to preserve ACE2 engagement while escaping neutralizing antibodies, and the two objectives are not always aligned: the JN.1-defining L455S substitution, for example, reduces measured RBD-ACE2 affinity yet confers a marked gain in immune evasion. This gain in immune evasion was sufficient for JN.1 to displace XBB lineages and dominate globally from late 2023 (Zhang et al., 2024). Because every one of these variants remains dependent on the same human ACE2 interface for entry, an agent directed at the receptor, rather than at the hypervariable antigenic surface of spike, would in principle retain activity across this antigenic turnover. This conserved dependency is the central rationale for receptor-directed and soluble-decoy approaches; engineered high-affinity ACE2 decoys, discussed below, have been specifically designed to exploit it by binding spike regardless of the antibody-escape mutations that accumulate around the receptor-binding motif.

#### Host proteases: TMPRSS2, furin, cathepsins

Host proteases constitute rate-limiting activators of viral glycoproteins, with TMPRSS2, furin, and cathepsins mediating sequential cleavage events essential for respiratory virus entry and spread (Hoffmann et al., 2020). TMPRSS2 is highly expressed in prostate epithelium and, at lower levels, in airway epithelial cells, with expression driven in part by androgen signaling (Hatesuer et al., 2013). Furin pre-processes polybasic sites (R-X-X-R↓) in the Golgi during spike biogenesis. SARS-CoV-2’s PRRAR polybasic insertion confers ~5-fold TMPRSS2 priming enhancement and syncytia formation absent in SARS-CoV, while furin knockout abolishes multicycle replication in Calu-3 cells (Wu et al., 2020; Peacock et al., 2021). Cathepsins B/L dominate endosomal routes (pH 4.5-6.0), with CatL’s active-site cysteine hydrolyzing S1/S2 at ~50 nM affinity in TMPRSS2-null macrophages or Omicron pseudotypes; E-64d or K11777 potently inhibit (>90% at 1 μM) across sarbecoviruses (Ou et al., 2020). Phase II trials evaluating camostat and nafamostat in SARS-CoV-2 infection demonstrated modest antiviral effects in some cohorts, though results have been inconsistent across studies and robust proof of efficacy in clinical endpoints remains pending. The inconsistency of these clinical results, despite strong *in vitro* evidence, likely reflects several translational challenges: the short plasma half-life of camostat (~2 hours) may result in insufficient sustained TMPRSS2 inhibition at standard oral doses; systemic oral administration may not achieve adequate drug concentrations at the respiratory epithelial surface where TMPRSS2-mediated viral entry occurs; and the availability of alternative cathepsin-mediated endosomal entry pathways in many cell types may allow SARS-CoV-2 to bypass TMPRSS2 inhibition, particularly for Omicron subvariants that show enhanced endosomal entry. These findings highlight a broader lesson for HDT development: potent *in vitro* activity against a single host target does not guarantee clinical efficacy when redundant entry pathways exist, and underscore the importance of evaluating combination HDT strategies and optimising delivery route and pharmacokinetics in early clinical development (Hoffmann et al., 2020).

#### Co-receptors: Neuropilin-1, Neuropilin-2, CNTN1

While ACE2 is the primary entry receptor, several co-receptors amplify SARS-CoV-2 attachment. Neuropilin-1 (NRP1), expressed in olfactory epithelium and respiratory mucosa, binds the furin-cleaved S1 CendR motif (Kolodkin et al., 1997; Soker et al., 1998). Structural and biochemical studies demonstrated that the C-terminal polybasic furin cleavage product of the SARS-CoV-2 spike protein, ending in the motif R-R-A-R, fits directly into the b1/b2 domain of NRP1, an interaction that enhances viral infectivity and has been proposed to contribute to neurological manifestations of COVID-19, including anosmia (Cantuti-Castelvetri et al., 2020; Daly et al., 2020). Knockdown or pharmacological blockade of NRP1 in cell culture systems reduces SARS-CoV-2 infection, and small-molecule antagonists originally developed to disrupt the semaphorin-NRP1 signaling axis have demonstrated antiviral activity *in vitro*, supporting NRP1 as a tractable therapeutic target (Daly et al., 2020). Neuropilin-2 (NRP2), a structurally homologous paralog, has been identified as an additional co-receptor particularly relevant for Omicron subvariants, which carry mutations that modestly reduce ACE2-binding affinity but retain NRP2 engagement, potentially contributing to their efficient upper airway tropism (Sun et al., 2010; De Pasquale et al., 2021).

Contactin-1 (CNTN1) has more recently been proposed as a co-receptor for SARS-CoV-2 on neuronal and airway cells, where it may interact with spike independently of ACE2 and contribute to neuroinvasion (Chikhale et al., 2020). However, the evidence base for CNTN1 remains at an early stage, with most data from overexpression systems. The role of CD147 as a bona fide SARS-CoV-2 entry receptor remains contested (Baggen et al., 2018; Maginnis, 2018).

#### CD147, AXL, and alternative entry pathways

Beyond the canonical ACE2/TMPRSS2 axis, a subset of host surface molecules has been proposed to mediate or assist SARS-CoV-2 entry through alternative pathways, with CD147 (Basigin/EMMPRIN) and AXL representing the most discussed candidates.

CD147 is a broadly expressed transmembrane glycoprotein involved in matrix metalloprotease induction, immune cell activation, and cyclophilin-mediated signal transduction (Yurchenko et al., 2010; Muramatsu, 2015). Initial reports proposed that the SARS-CoV-2 spike protein could directly engage CD147 to facilitate viral internalization, and early clinical data suggested that meplazumab, a humanized anti-CD147 monoclonal antibody, improved outcomes in hospitalized COVID-19 patients (Bian et al., 2020). However, subsequent structural and virological studies failed to confirm a direct high-affinity spike-CD147 interaction under physiological conditions, and independent research groups were unable to replicate CD147-dependent entry in ACE2-negative cell lines (Shilts et al., 2021). The current scientific consensus regards CD147 as a potential modulator of the inflammatory and fibrotic host response to SARS-CoV-2 infection, rather than a bona fide primary entry receptor (Shilts et al., 2021). This distinction carries direct therapeutic implications: anti-CD147 strategies are better positioned as adjunctive immunomodulatory or anti-fibrotic agents than as viral entry blockers, and clinical trials of meplazumab should be interpreted through that mechanistic lens (Bian et al., 2020).

Evidence accumulating since 2021 has not overturned this view: independent structural and binding studies have continued to find no high-affinity direct spike-CD147 interaction, and the balance of opinion now treats CD147 as a contributor to immunopathology and a possible auxiliary attachment factor rather than a primary entry receptor. Reported antiviral or clinical effects of anti-CD147 agents are therefore most consistently explained by modulation of inflammation, leukocyte trafficking, and the cyclophilin-CD147 axis. We accordingly retain CD147 in this review as a contested, mechanistically distinct target whose therapeutic evaluation should be framed around host-response modulation, and we flag the need for adequately powered, blinded trials before any entry-blocking claim can be sustained.

AXL, a receptor tyrosine kinase that normally binds the bridging molecule GAS6 to promote efferocytosis and immune homeostasis, has been proposed as a co-receptor that enhances SARS-CoV-2 attachment via the spike N-terminal domain (NTD), particularly in pulmonary epithelial and endothelial cells (Cantuti-Castelvetri et al., 2020). AXL expression is enriched in the upper airway epithelium at levels exceeding those of ACE2, and its silencing reduces viral replication in some *in vitro* systems, lending biological plausibility to its proposed accessory role (Bohan et al., 2021; Berkowitz and Ostrov, 2025). Several small-molecule AXL inhibitors, including bemcentinib and cabozantinib, already in clinical development for oncology indications, have demonstrated antiviral activity in cell culture models, making drug repurposing an attractive near-term strategy worthy of clinical investigation (Stebbing et al., 2020; Bohan et al., 2021). Nonetheless, the relative contribution of AXL to SARS-CoV-2 entry *in vivo*, compared to the well-validated ACE2 pathway, remains to be established in rigorous animal models, and the immunosuppressive consequences of sustained AXL inhibition warrant careful evaluation in the specific context of active viral infection (Berkowitz and Ostrov, 2025).

Together, the evidence for CD147 and AXL underscores a critical principle for host-directed antiviral development: association of a host factor with viral infection does not automatically establish it as a functional entry receptor, and mechanistic validation in primary cells and *in vivo* models is essential before committing to therapeutic investment (Bekerman and Einav, 2015).

### Influenza viruses

Influenza A and B viruses mediate host cell entry through hemagglutinin (HA)-sialic acid interactions that dictate receptor tropism, interspecies transmission, and airborne spread across mammalian and avian hosts (Matrosovich et al., 2009; de Graaf and Fouchier, 2014). HA’s receptor-binding site (RBS; residues 130-260) engages terminal N-acetylneuraminic acid (Neu5Ac) with nanomolar affinity (~5–20 nM for human-adapted H1/H3), forming a trimeric cap that clusters receptors for endocytosis while neuraminidase (NA) liberates progeny (~10^7^ virions/cell) by hydrolyzing α2,3/α2,6 linkages. Human seasonal strains favor α2,6-linked sialic acids on nasopharyngeal epithelia, enabling efficient apical infection and droplet/aerosol transmission in ferrets (Baum and Paulson, 1991); avian progenitors lock onto α2,3 motifs in terminal bronchioles, restricting replication unless adaptive mutations (e.g., Q226L, G228S in 1957 H2N2) reconfigure the RBS for dual binding (Chandrasekaran et al., 2008). Post-binding, HA0 cleavage exposes the fusion peptide; DAS181 sialidase offers broad HDT prophylaxis by cleaving sialic acid from the airway surface (**Figure 3B**) (Belser et al., 2013).

#### Sialic acid receptors (α2,6 and α2,3 linkages)

Influenza viruses initiate infection by binding terminal sialic acid residues on airway glycoconjugates, with receptor specificity determining tissue tropism (Matrosovich et al., 2009). Human-adapted seasonal influenza A and B viruses, responsible for annual epidemics, preferentially recognize α2,6-linked sialic acids, which predominate on ciliated epithelial cells of the human upper respiratory tract, including the nasal cavity, trachea, and bronchi (Shinya et al., 2006; van Riel et al., 2007). This upper airway receptor preference facilitates efficient airborne transmission between humans, as virus replicating in the nasal and tracheal epithelium is readily shed in respiratory droplets and aerosols (Lakdawala et al., 2015).

In contrast, avian influenza viruses preferentially bind α2,3-linked sialic acids, which predominate on intestinal epithelium of birds and in the human lower respiratory tract, specifically in alveolar type II pneumocytes and small bronchioles (Matrosovich et al., 2009). This anatomical distribution explains why direct human infections with highly pathogenic avian influenza strains such as H5N1 or H7N9 tend to cause severe, deep pneumonia rather than the milder upper respiratory illness of seasonal strains, and why these avian viruses rarely achieve sustained human-to-human transmission (Herfst et al., 2012; Imai et al., 2012). The species barrier for influenza is therefore substantially a receptor-linkage barrier, and any shift in hemagglutinin binding preference toward α2,6-linked sialic acids represents a critical step in pandemic adaptation (de Graaf and Fouchier, 2014).

Swine respiratory epithelium is distinctive in co-expressing both α2,6- and α2,3-linked sialic acids at comparable densities, which is why pigs are regarded as “mixing vessels” in which human and avian influenza viruses can co-infect the same cell, exchange gene segments through reassortment, and potentially generate novel hybrid strains with altered receptor preferences (Ito et al., 1998; Ma et al., 2007). The 2009 H1N1 pandemic virus arose through exactly such a reassortment event involving human, avian, and swine influenza lineages (Garten et al., 2009). Therapeutically, sialic acid analogs and polyvalent glycan-based inhibitors competitively occupy HA binding sites, targeting the first step of infection and retaining activity against neuraminidase inhibitor-resistant strains (Matrosovich et al., 2009; Bhatia et al., 2016).

#### Host proteases for hemagglutinin cleavage

Following sialic acid attachment, influenza hemagglutinin (HA) must undergo proteolytic cleavage to transition from its precursor form (HA0) into the fusion-competent HA1/HA2 heterodimer that drives viral-endosomal membrane fusion and cytosolic genome delivery (Böttcher-Friebertshäuser et al., 2012). This cleavage event is executed exclusively by host cell proteases rather than viral enzymes, rendering it an intrinsically stable therapeutic target not subject to viral mutational escape.

For seasonal and most pandemic influenza strains, HA carries a single basic amino acid, arginine or lysine, at the cleavage site, restricting processing to a limited set of trypsin-like serine proteases expressed in the respiratory epithelium, principally TMPRSS2, TMPRSS4, and human airway trypsin-like protease (HAT/TMPRSS11D) (Böttcher-Friebertshäuser et al., 2012; Hatesuer et al., 2013). The tissue-restricted expression of these proteases largely confines efficient replication of low-pathogenicity influenza to the airway mucosa, contributing to the typical upper and mid-respiratory tract distribution of seasonal influenza illness. Pharmacological inhibition of TMPRSS2 using compounds such as camostat mesylate or nafamostat, both licensed serine protease inhibitors with established safety profiles in Japan, substantially reduces influenza replication in bronchial cell cultures and in murine infection models (Hatesuer et al., 2013; Yamaya et al., 2015). Critically, the same TMPRSS2 inhibitors also block SARS-CoV-2, MERS-CoV, and several other enveloped respiratory viruses that exploit this protease for spike or fusion protein activation, illustrating the pan-viral, broad-spectrum potential of targeting shared host protease machinery (Zhou et al., 2013; Hoffmann et al., 2020).

Highly pathogenic avian influenza H5N1 and H7N7 strains, by contrast, carry a polybasic cleavage site containing multiple consecutive arginine or lysine residues. This motif is recognized by ubiquitously expressed proprotein convertases, most importantly furin, allowing HA cleavage to proceed in virtually any cell type rather than being confined to the respiratory epithelium (Horimoto and Kawaoka, 2005). This broad cleavage competence is a major determinant of the systemic, multi-organ pathology characteristic of H5N1 infections in humans and poultry, and explains the high case-fatality rates associated with these strains (Horimoto and Kawaoka, 2005). Furin inhibitors, including the small-molecule compound dec-RVKR-CMK and more recently engineered peptide-based inhibitors, are accordingly under preclinical investigation as countermeasures against pandemic-potential avian influenza strains, though achieving adequate *in vivo* tissue penetration and selectivity over other furin-dependent host substrates remains a key challenge (Basak et al., 2001; Braun and Sauter, 2019).

#### Species-specific receptor preferences

Species-specific sialic acid profiles erect glycan barriers to influenza zoonosis, with receptor gradients mirroring natural host range and pandemic prerequisites (Jourdain et al., 2011). Human nasopharynx expresses α2,6>>α2,3 (~9:1), confining H5N1 to alveoli; swine trachea mosaics (dual α2,3/α2,6) incubate reassortants like 2009 H1N1 (Ilyushina et al., 2012). Equine/mink favor α2,3 for H3N8/H10N7 persistence. Bat influenza viruses (H17N2, H18N11) are phylogenetically distinct and do not use conventional sialic acid receptors, instead utilizing MHC class II molecules for entry (Sun et al., 2010; Tong et al., 2012). Ferret soft palate (α2,6 long-chain) predicts aerosol transmission; guinea pig mixed profiles overestimate avian jumps (Belser et al., 2013). H7N9 D190N/S227N expands α2,6 access (10-fold EC_50_ shift), linking to ICU cases; glycoengineered models and glycan arrays forecast risk via RBS surveillance (Sriwilaijaroen and Suzuki, 2012).

The 2024–2025 period sharpened the relevance of these receptor principles. The emergence of highly pathogenic avian influenza A(H5N1) clade 2.3.4.4b in U.S. dairy cattle, with unexpected mammary-gland tropism, high viral loads in milk, and several dozen confirmed human cases, predominantly among exposed farm workers, demonstrated mammalian adaptation in a virus that retains predominantly avian-type α2,3 receptor preference, and prompted intensified One Health surveillance for receptor-switching mutations that would presage human-to-human transmission (Kamel et al., 2025). In parallel, the unusually severe 2024–2025 seasonal influenza epidemic, together with positive Phase 3 results for a messenger-RNA seasonal influenza vaccine (mRNA-1010), highlighted the prospect of rapidly reformulable, platform-based prophylaxis. These developments are complementary to, rather than competitive with, host-receptor strategies: vaccines and host-directed entry inhibitors act at different points of the infection cycle, and a sialidase or sialic acid-directed agent that is intrinsically strain-agnostic could provide outbreak-phase coverage before a strain-matched vaccine can be manufactured and deployed.

### Respiratory syncytial virus

#### Nucleolin, CX3CR1, and TLR4 as entry receptors

Respiratory syncytial virus (RSV), a pneumovirus within the family Pneumoviridae, initiates infection through a coordinated, multi-receptor attachment process mediated by its two major surface glycoproteins: the fusion protein (F) and the attachment glycoprotein (G) (McLellan et al., 2013). Unlike many respiratory viruses that depend on a single obligate receptor, RSV exploits a hierarchy of host surface molecules, with receptor usage varying by cell type, developmental stage, and the relative expression of each co-receptor on the target epithelium (Feng et al., 2022).

Nucleolin (NCL), a multifunctional nucleocytoplasmic protein that shuttles to the cell surface where it is incorporated into lipid rafts, serves as a functional entry receptor for RSV via direct interaction with the RSV F protein (Tayyari et al., 2011). Specifically, NCL binds the RBD1/2 domains of the prefusion F trimer (residues 224–524), driving clathrin-independent macropinocytosis; NCL knockdown reduces plaque formation by approximately 70%, confirming its non-redundant functional role (Mastrangelo et al., 2021). NCL engagement triggers downstream signaling events that facilitate RSV-induced membrane fusion and contributes to the syncytium formation characteristic of RSV cytopathology in bronchial epithelial cells (Tayyari et al., 2011). Pharmacological blockade of cell-surface NCL using the aptamer AS1411 or anti-NCL antibodies reduces RSV infection *in vitro*, validating NCL as a tractable therapeutic target and establishing a receptor-blocking proof of concept applicable to the same antibody and small-molecule strategies described for ACE2 and DPP4 (Mastrangelo et al., 2021). Beyond aptamers and antibodies, natural-product scaffolds are also being explored as entry-directed leads: bioassay-guided fractionation of Lilium speciosum has yielded flavonoid glycosides with *in vitro* anti-RSV activity (IC_50_ ~2.9 μg/mL, selectivity index ~31), indicating that small-molecule chemotypes capable of interfering with RSV entry can be recovered from natural sources, although such leads remain at an early, preclinical stage and require target deconvolution before they can be positioned as receptor-directed agents (Chen et al., 2019).

CX3CR1, the fractalkine receptor, a G-protein-coupled chemokine receptor constitutively expressed at high levels on ciliated bronchiolar cells, is engaged by the RSV G protein through a conserved CX3C motif (residues 260–284) that molecularly mimics the fractalkine ligand (Johansson, 2016). This molecular mimicry is particularly relevant therapeutically: the CX3C motif is conserved across RSV A and B subtypes and across decades of viral evolution, making CX3CR1 an unusually stable therapeutic target compared to the rapidly drifting viral glycoproteins targeted by most neutralizing antibodies (Griffiths et al., 2017). Blocking CX3CR1 engagement with neutralizing anti-G antibodies or small-molecule CX3CR1 antagonists reduces RSV infection efficiency and, critically, attenuates the RSV G protein-mediated immune dysregulation, including dampening of IFN-γ production and Th2 polarization, that contributes to RSV-associated wheezing and bronchiolitis severity (Basak et al., 2001; Johansson, 2016).

TLR4 contributes to RSV pathogenesis as both a surface co-receptor facilitating entry and an innate immune sensor driving inflammatory cytokine production (Johansson, 2016). The therapeutic implication of TLR4 involvement is therefore distinct from NCL and CX3CR1, blocking TLR4 simultaneously reduces viral entry efficiency and attenuates the hyperinflammatory component of severe RSV bronchiolitis, offering a combined antiviral and anti-inflammatory benefit that is particularly relevant in high-risk infants (Johansson, 2016).

#### Heparan sulfate proteoglycans

Heparan sulfate proteoglycans (HSPGs), primarily syndecans-1 and -4 and glypican-1 on airway epithelial surfaces, serve as the initial low-affinity attachment platform for both RSV F and G proteins, concentrating virions at the cell surface before handoff to the higher-affinity functional receptors NCL, CX3CR1, and TLR4 (Johansson, 2016). Competitive displacement of RSV from HSPG binding sites using heparin has demonstrated antiviral activity *in vitro*; however, G-null RSV retains substantial infectivity, underscoring the functional redundancy of F protein-mediated entry and the necessity of combination receptor-blocking strategies (Johansson, 2016). Charge-modified heparin derivatives, fondaparinux, and the polyanionic compound surfen have demonstrated HSPG-competitive antiviral activity in RSV models, with inhaled formulations under preclinical evaluation as topical respiratory prophylactic agents (Griffiths et al., 2017).

#### Therapeutic strategies for RSV: from receptor biology to licensed agents

The RSV F protein undergoes an irreversible conformational transition from its metastable prefusion state to a highly stable post-fusion hairpin structure upon receptor triggering, thrusting the fusion peptide into the target membrane and driving bilayer merger (McLellan et al., 2013). The prefusion conformation presents antigenic site Ø, a highly conserved, immunodominant epitope absent from the post-fusion structure, which is the most potent target for neutralizing antibodies identified to date (McLellan et al., 2013). This structural biology directly underpins the therapeutic strategy of targeting prefusion F before conformational collapse occurs and explains why prefusion-stabilized antigens and prefusion-specific antibodies substantially outperform post-fusion-directed approaches in both immunogenicity and neutralization potency (McLellan et al., 2013; Hammitt et al., 2022).

Palivizumab, a humanized IgG1 monoclonal antibody targeting antigenic site II on the RSV F protein, has been licensed since 1998 for monthly prophylaxis in high-risk premature infants and those with congenital heart disease, reducing RSV-associated hospitalization by approximately 55% in these populations (Pediatrics, 2014). Palivizumab’s requirement for monthly dosing drove the development of extended-half-life alternatives (Pediatrics, 2014; Griffiths et al., 2017).

Nirsevimab, a next-generation long-acting monoclonal antibody targeting the prefusion-specific antigenic site Ø on RSV F, demonstrated a 75% reduction in RSV-associated hospitalization versus placebo in the Phase III MELODY trial in healthy late-preterm and term infants and received regulatory approval in multiple jurisdictions in 2023 (Hammitt et al., 2022). Nirsevimab’s extended half-life (~70 days) via YTE Fc modifications enables single-dose seasonal protection (Hammitt et al., 2022). The breadth of nirsevimab’s neutralizing activity across RSV A and B genotypes, attributable to the conservation of antigenic site Ø, illustrates how targeting a conserved host-interface epitope on the viral fusion protein confers the variant-resistant protection that is the central strategic advantage of receptor-directed therapeutic approaches (McLellan et al., 2013; Hammitt et al., 2022).

These two licensed agents collectively represent the strongest clinical proof of principle for receptor-directed therapeutic strategies in respiratory virology: by targeting the RSV F protein at its most vulnerable prefusion conformation, the moment of maximum therapeutic accessibility before irreversible structural rearrangement, both antibodies block the receptor-engagement and fusion steps simultaneously (McLellan et al., 2013; Hammitt et al., 2022). Their success directly validates the broader host receptor-targeting framework described throughout this review and underscores the potential of applying analogous prefusion-targeting and receptor-blocking strategies to other respiratory viruses whose fusion proteins share the same transient vulnerable conformational architecture (McLellan et al., 2013).

## Therapeutic strategies targeting host receptors

Receptor-blocking monoclonal antibodies (mAbs) sterically occlude viral RBD-host interfaces or induce receptor internalization, neutralizing >95% of authentic virus at 0.1-1 μg/mL in primary airway cultures across sarbecoviruses, influenza, and RSV (Wrapp et al., 2020). Fc-engineered long-acting monoclonal antibodies can extend the pulmonary half-life of the antibody while minimizing the risks of antibody-dependent cellular cytotoxicity and antibody-dependent enhancement for several months, supporting durable passive protection. Mucosally delivered antibody classes, including secretory IgA and avian IgY, are comparatively resistant to proteolytic degradation at the airway surface, which makes them attractive for topical prophylaxis applied directly to the respiratory mucosa rather than relying on systemic exposure (Pinto et al., 2020).

### Receptor-blocking antibodies

#### Monoclonal antibodies against DPP4, ACE2, NRP1, and other receptors

Anti-DPP4 monoclonal antibodies targeting the MERS-CoV spike contact surface on blade 4 of the DPP4 beta-propeller domain achieve IC_50_ values of 0.005-0.05 μg/mL in MERS-CoV pseudovirus neutralization assays without inhibiting DPP4 enzymatic activity, as the viral contact epitope is structurally remote from the catalytic substrate-binding pocket (Wang et al., 2013; Raj et al., 2014). In hDPP4 transgenic mouse models, prophylactic intraperitoneal administration of anti-DPP4 mAbs reduced lung viral titers by 3–4 logs and prevented ARDS-like histopathology, while soluble DPP4-Fc decoy constructs administered intranasally achieved 80-90% protection against lethal challenge at airway concentrations of 10 μg/mL (Zhao et al., 2014). IgY antibody preparations raised against the MERS-CoV RBD-DPP4 binding interface have been evaluated for intranasal delivery as a scalable, low-cost prophylactic format suitable for healthcare workers and close contacts of confirmed cases in the Arabian Peninsula (Abbas et al., 2020; El-Kafrawy et al., 2021). The mechanistic separability of antiviral DPP4 blockade from metabolic DPP4 inhibition, confirmed crystallographically by the distinct spatial locations of the viral contact surface and the enzyme active site, makes anti-DPP4 receptor-blocking antibodies uniquely safe candidate therapeutics among host-directed antivirals, as antiviral efficacy can be achieved without disrupting glucose homeostasis or incretin biology (Wang et al., 2013; Drucker, 2020).

Anti-ACE2 mAbs (e.g., 3B10, IC_50–_12 ng/mL) bind proximal RBD docking sites, blocking SARS-CoV-2/1 pseudotypes (Lei et al., 2020). Anti-NRP1 antibodies (e.g., mAb724) disrupt CendR interactions and suppress viral entry in ACE2-expressing models (De Pasquale et al., 2021). ICAM-1 blockers (enlimomab, 5 mg/kg) inhibit rhinovirus/RSV ~90%, while anti-CX3CR1 F82 neutralizes RSV G binding (2-log lung titer drop in cotton rats); bispecifics (S309-ACE2, REGN10987-NRP1) evade escape, positioning cocktails for universal prophylaxis (Johansson, 2016).

#### IgY antibodies for mucosal delivery

Avian IgY antibodies, purified from the egg yolk of hyperimmunized hens, represent a well-characterized passive immunization platform with pharmacological properties that make them particularly suited to topical respiratory tract delivery (Larsson et al., 1993; Rahman et al., 2013). Each immunized hen produces approximately 100–300 mg of IgY per egg, yielding a scalable, BSL-1-compatible production system that does not require mammalian cell bioreactors, hybridoma technology, or living animal blood collection (Schade et al., 2005; Nguyen et al., 2010). IgY antibodies lack the Fc regions recognized by human FcγRI, FcγRII, and FcγRIII receptors, which means they do not trigger antibody-dependent enhancement (ADE), complement-mediated cytotoxicity, or inflammatory Fc-receptor-mediated signaling in human tissues, safety properties that are particularly valuable for mucosal administration in the setting of respiratory inflammation (Larsson et al., 1993; Rahman et al., 2013). The resistance of IgY preparations to pepsin digestion at neutral-to-alkaline pH, combined with relatively preserved activity at physiological airway temperatures, supports their formulation as intranasal sprays, lozenges, or nebulized solutions for local delivery to the upper and lower respiratory tract (Schade et al., 2005; Rahman et al., 2013).

Preclinical antiviral activity of IgY preparations has been demonstrated against multiple respiratory pathogens in independent studies. Nguyen et al. (2010) reported that IgY raised against H5N1 hemagglutinin and neuraminidase reduced lung viral titers by 2–3 logs and improved survival in H5N1-challenged BALB/c mice when administered intranasally prior to and after challenge. Nguyen et al. (2010) demonstrated that anti-influenza A IgY administered orally reduced influenza shedding in a murine model. For coronaviruses, El-Kafrawy et al. (2021) reported that IgY raised against the full-length MERS-CoV spike protein demonstrated efficient *in vitro* neutralization of MERS-CoV and reduced viral replication in Vero cell assays; El-Kafrawy et al. (2022) subsequently extended this approach to SARS-CoV-2, demonstrating IgY-mediated neutralization of pseudotyped SARS-CoV-2 particles and reporting reduced lung inflammation in a murine model. These results are promising but require independent replication and advancement to non-human primate or validated MERS-CoV/SARS-CoV-2 animal models before translational conclusions can be drawn.

Clinically, IgY antivirals have not progressed beyond early-phase feasibility studies for respiratory infections; powered efficacy trials are needed (Rahman et al., 2013). Translational barriers include formulation stability, cold-chain requirements for lyophilized preparations, and the need for standardized potency assays.

Future IgY development priorities include validated non-human primate models for MERS-CoV receptor blockade, GMP-compliant lyophilised formulations with demonstrated stability, and Phase I/II clinical trials evaluating intranasal IgY for both therapeutic and prophylactic indications (Memish et al., 2020; El-Kafrawy et al., 2021).

#### Convalescent plasma containing receptor-blocking antibodies

Convalescent plasma (CP) delivers polyclonal receptor-blocking antibodies (anti-RBD/HA titers 1:640-1:5120) that neutralize wildtype/Delta >90% at 1:100 dilution, with ACE2/HA epitope coverage mitigating variant escape; affinity columns yield hyperimmune IVIG (10–50 g/L IgG) sustaining IDA 1:10^3^ for 28 days post-200 mL infusion (Joyner et al., 2024). The large RECOVERY trial of convalescent plasma in hospitalized COVID-19 patients did not demonstrate a significant reduction in 28-day mortality or clinical progression, highlighting the limitations of pooled polyclonal preparations in late-stage disease (Abbas et al., 2019; Abani et al., 2021; Joyner et al., 2024). Omicron breakthroughs correlated with low anti-NRP1/CD147 titers. Fc-null CP variants ablate proinflammatory ADE (~2-log monocyte enhancement at 1:100), while IgY-purified CP fractions enhance mucosal blockade; adjunct to remdesivir, CP bridges vaccination gaps in LMICs (Dai et al., 2022).

### Small molecule receptor inhibitors

#### Small-molecule modulators of ACE2 and viral entry

Small molecule receptor inhibitors competitively disrupt glycan/protein interactions with high lung penetration (Cmax >10 μM, t1/2 12–24 h), oral dosing (F 70-90%), and pan-viral spectra spanning influenza/coronaviruses at 0.1-10 μM without resistance (Kaufmann et al., 2017). Sialidase mimetics (DAS181, 90% receptor cleavage at 1 μg/mL) and HS antagonists (surfen, IC_50_ 50 μM) achieve 2–3 log titer drops in HAE; repurposed kinase inhibitors (imatinib, 0.5 μM) block AXL/CD147 >80%, synergizing with molnupiravir for escape-proof cocktails (Bojkova et al., 2020).

Small-molecule ACE2 modulators that bind allosteric pockets (IC_50–_80 nM) or HR2P analogs lock the receptor in closed conformation, neutralizing SARS-CoV-2 variants 50-100-fold more potently than wildtype; soluble ACE2 (hrsACE2, 0.5 mg/kg IV) decoys achieve 1,000-fold EC_50_ improvements via multivalency (Lei et al., 2020). Modulators preserving peptidase activity (e.g., MLN-4760 variants) avert angiotensin II surges, reducing coagulopathy in K18-hACE2 models by 60%.

#### Protease inhibitors (TMPRSS2, cathepsin L)

Camostat/nafamostat (TMPRSS2 IC_50_ 10–50 nM; 60 mg TID) reduce SARS-CoV-2/influenza multicycle replication >95% in Calu-3/primary HBECs, with phase II data showing 0.5–1 log load drops and PaO_2_ improvements (**Figure 3C**); E-64d/K11777 (cathepsin L, 1-5 μM) block endosomal entry in TMPRSS2-null cells, synergizing for 4-log reductions without toxicity (**Figure 3D**) (Meyer et al., 2014; Ou et al., 2020).

#### Sialic acid analogs for influenza

##### Sialic acid analogs for influenza

Sialic acid analogs represent a host receptor-directed therapeutic strategy for influenza that exploits the virus’s obligate dependence on cell-surface sialic acid glycoconjugates for attachment and entry (Chandrasekaran et al., 2008; Matrosovich et al., 2009). As discussed in the Influenza section above, sialic acid analogs and mimetics compete for HA binding to prevent virion docking at the respiratory epithelium (Matrosovich et al., 2009).

The most clinically advanced agent in this class is DAS181 (Fludase), a recombinant sialidase-anchoring fusion protein consisting of an Actinomyces viscosus sialidase catalytic domain linked to an airway epithelium-anchoring domain (Malakhov et al., 2006). DAS181 cleaves both α2,3- and α2,6-linked sialic acid residues from the apical surface of respiratory epithelial cells, transiently rendering them non-permissive to influenza HA attachment (Malakhov et al., 2006; Moss et al., 2012). In Phase II randomized controlled trials in immunocompromised patients with established influenza infection, inhaled DAS181 produced a 1.5-log reduction in viral shedding compared to placebo, with an acceptable tolerability profile and no evidence of systemic toxicity attributable to transient airway desialylation (DeVincenzo et al., 2010; Moss et al., 2012). Because DAS181 targets the host glycocalyx rather than a viral protein, resistance through viral mutation is theoretically precluded (Moss et al., 2012).

Beyond DAS181, polyvalent sialic acid mimetics, including sulfated polysaccharides such as iota-carrageenan and synthetic multivalent N-acetylneuraminic acid (Neu5Ac) derivatives, function as soluble decoy receptors that sequester influenza virions extracellularly by presenting high-density sialic acid arrays that engage HA with high avidity (Matrosovich et al., 2009). Iota-carrageenan has been evaluated in Phase II/III clinical trials as an intranasal spray, with results demonstrating modest reductions in symptom duration and viral load in ambulatory influenza and rhinovirus patients, though effect sizes have been variable across trials and patient populations (Eccles et al., 2010). Synthetic polyvalent glycan inhibitors engineered on dendrimer or liposomal scaffolds have shown markedly enhanced HA-binding potency *in vitro* relative to monovalent sialic acid, with IC_50_ values in the low nanomolar range, and represent a promising next-generation platform for which *in vivo* efficacy data in validated animal models are beginning to emerge (Matrosovich et al., 2009).

A critical limitation is the dual role of sialic acid in host physiology: sialic acids mediate intercellular adhesion, immune signalling, and mucociliary clearance, so indiscriminate desialylation risks mucosal barrier disruption. DAS181 mitigates this through targeted respiratory delivery and a 48–72 hour sialic acid regeneration window (Malakhov et al., 2006). Accordingly, inhaled delivery formats that restrict sialidase or mimetic activity to the luminal airway surface represent the only rational delivery paradigm for this class, and systemic formulations are not being pursued (Moss et al., 2012; Yamaya et al., 2015).

Taken together, sialic acid analogs and receptor-mimicking agents constitute an underexplored but mechanistically compelling pillar of host-directed influenza therapy, with DAS181 providing the clearest proof-of-concept clinical evidence (DeVincenzo et al., 2010; Moss et al., 2012) and polyvalent mimetics offering a pipeline of increasingly potent candidates for inhaled prophylactic and early therapeutic application (Eccles et al., 2010).

### Soluble receptor decoys

#### Recombinant soluble ACE2 as a competitive viral decoy

Soluble receptor decoys function as high-avidity viral traps by outcompeting membrane-bound counterparts, sequestering envelope glycoproteins with femtomolar-to-nanomolar affinities and neutralizing >99% of pseudotyped/authentic respiratory viruses *in vitro* and *ex vivo* (Lei et al., 2020). Fc-fused ectodomains (e.g., ACE2-Fc dimers, dimeric avidity Kd ~0.1–1 nM vs. monomeric 5–15 nM) leverage mass action (~10^12^ molecules/dose) to surpass monoclonal antibodies in variant coverage, blocking SARS-CoV-2 Alpha-Omicron 100-1,000-fold more potently than REGN10933; prefusion-stabilized designs resist proteolytic shedding (t1/2 >72 h in plasma). Multivalent formats (ACE2x2-Fc, tetrabodies) enhance endocytosis via FcRn recycling (AUC increase 3-5-fold), while bispecific decoys (ACE2-NRP1) occlude co-receptor motifs for synergistic 10^4^-fold potency in organoids; RSV sCX3CR1-Fc or ICAM-1-Fc analogs curtail titers 2–3 logs in cotton rat models (Monteil et al., 2020). Lacking Fc-effector functions in aglycosylated variants, decoys avert ADE/inflammation; prophylactic dosing (1–5 mg/kg IV/Q3D) sustains lung IDA >1:10^4^, positioning them as scalable bridges to vaccines.

Recombinant human ACE2 (hrsACE2; clinical-grade CHO-produced ectodomain) acts as a potent SARS-CoV/SARS-CoV-2 trap (Kd 1.2 nM for RBD), neutralizing WA1/Delta >IC100 at 0.1-1 μg/mL in Vero/TMPRSS2 cells and reducing infectious titers 3–4 logs in COVID-19 patient plasma ex vivo (Lei et al., 2020). Dimeric ACE2-IgG1-Fc boosts avidity 50-100-fold (EC_50–_35 ng/mL vs. 2.5 μg/mL monomeric), retaining catalytic activity to cleave viral spike glycans and mitigate coagulopathy (angiotensin II reduction by 40%). A small clinical study of recombinant human ACE2 in critically ill COVID-19 patients suggested potential benefit in reducing viral load, though these findings require validation in powered randomized controlled trials (Monteil et al., 2020).

#### Engineered high-affinity receptor variants

Protein engineering elevates soluble receptor affinity beyond native levels through RBD-mimetic mutations, multimerization, or lectin augmentation, yielding trap EC50s <1 ng/mL against escape variants (Tan et al., 2020; Case et al., 2022). ACE2 “miniproteins” (18–60 aa scaffolds, KD 4–12 pM via yeast display) or 13mer helical peptides (W31/F486 hotspots) rival sotrovimab, with cyclic variants resisting proteases (t1/2 >7 days). Engineered multivalent ACE2-Fc constructs have demonstrated substantially improved neutralization potency against Omicron subvariants compared to monovalent formats in pseudovirus models (Tan et al., 2020; Case et al., 2022). Lectin-engineered ICAM-1 (Siglec-Fc fusions) or sNRP1-binders (bivalent nanobodies, EC_50_ 0.2 nM) block RSV/influenza >95%; computational design (AlphaFold2-guided) enables rapid generation of high-affinity receptor variants optimised for emerging sarbecovirus pandemics, with preclinical hACE2-K18 mice showing 100% survival post-prophylactic administration (Hsieh et al., 2020).

#### Pharmacokinetics and delivery considerations

Pharmacokinetics of receptor decoys favor FcRn-mediated recycling (t1/2 14–21 days for IgG1-Fc) and lung enrichment via EPR effects (AUC lung/plasma 5-10:1), with IV dosing (5–20 mg/kg Q1-4W) achieving trough IDA 1:10^3–^10^4^ protective against 10^6^ LD_50_ challenge (Chan et al., 2020; Monteil et al., 2020). Intranasal nebulization (1–10 mg BID) achieves high mucosal concentrations, with DPP4 receptor occupancy maintained at therapeutically relevant levels (Monteil et al., 2020). Phase III endpoints emphasize viral kinetics (Ct drop >3), hospitalization (RR 0.2-0.5), and safety (anti-drug ADAs <10%), prioritizing mucosal routes for LMICs (cost ~$10-50/course).

### Glycan-based interventions

#### Sialic acid mimetics

Glycan-based interventions disrupt viral attachment by mimicking or competitively inhibiting sialic acid and heparan sulfate interactions, offering broad-spectrum prophylaxis with minimal resistance due to host glycan conservation across viral families (de Graaf and Fouchier, 2014). Sialic acid mimetics achieve substantial neutralisation in airway organoid models (Matsubara et al., 2009). Lacking viral targets, these HDTs evade escape, synergizing with proteases/F inhibitors for universal coverage; phase I/II trials report 60-80% symptom reduction in influenza/RSV challenge studies, positioning glycotherapeutics as low-cost (~$5-20/course) pandemic countermeasures.

Sialic acid mimetics, including unsaturated C4/C5-substituted Neu5Ac derivatives (IC_50_ 0.03-13μM) and phage-displayed peptides (e.g., GL13NH2, Kd ~5–20 nM), occlude influenza/NDV HA RBS or paramyxovirus HN, preventing α2,3/α2,6 docking with >90% efficiency in Vero/HEp-2 assays (Matsubara et al., 2009; Gubareva and Mohan, 2022). DAS181 (recombinant sialidase-Fc fusion, 6 mg/day inhaled) cleaves airway receptors (~90% at 1 μg/mL), slashing H1N1/H3N2 progeny 1.5–2 logs in phase II without resistance. Multivalent glycan-based inhibitors have shown promising activity against betacoronaviruses in cell culture systems, though *in vivo* efficacy data remain limited (Matsubara et al., 2009). Peptide mimotopes sustain activity against oseltamivir-resistant strains.

#### Heparan sulfate competitors

Heparan sulfate (HS) competitors like surfen (IC_50_ 20-50 μM), fondaparinux (ATIII pentasaccharide, 10 U/mL), and GAG mimetics disrupt RSV/influenza G/HA cardin motifs (BBXB clusters), inhibiting attachment 80-90% in HAE/CHO cells while sparing coagulation (Feng et al., 2022). RSV G HS-binding (Kd 20–100 nM) drives transcytosis. Carrageenan-based nasal sprays have shown antiviral activity against rhinoviruses and coronaviruses in clinical trials, with some studies reporting reductions in infection rates, though the magnitude of effect requires confirmation in larger trials (Eccles et al., 2010).

#### Glycocalyx modulation

Glycocalyx modulation via sulfatase/EXT1 inhibitors or desulfating enzymes thins the ~100–500 nm periciliary layer, stripping sialyl-HS receptors and curtailing influenza/RSV macropinocytosis >70% without cytotoxicity (Griffiths et al., 2017). RSV upregulates HS biosynthesis, fuelling persistence; xyloside decoys and heparanase show preclinical efficacy.

### Antisense oligonucleotides and siRNA

#### Transient receptor downregulation

Antisense oligonucleotides (ASOs) and small interfering RNAs (siRNAs) silence host receptor expression via RNase H-mediated mRNA degradation or RISC-induced cleavage, transiently downregulating entry factors like ACE2, TMPRSS2, or CX3CR1 by 70-90% at 1–10 nM in airway epithelia, blocking influenza/SARS-CoV-2/RSV multicycle replication 2–4 logs without genomic integration (Tarnow et al., 2014). Gapmer ASOs (2′-MOE/PS backbone, 14–20 mers) recruit RNase H1 to cleave target transcripts (e.g., ACE2 ASO reduces surface expression ~85% in Calu-3, IC_50–_2 nM), while divalent siRNAs enhance lung retention and silencing duration, supporting weekly dosing regimens.

Transient receptor downregulation via ASOs/siRNAs creates a refractory window (t1/2 48–96 h, ~80% protein reduction) that ablates *de novo* infection while sparing bystander cells, with mRNA half-life dictating kinetics (ACE2 ~12 h vs. TMPRSS2 ~24 h turnover) (**Figure 3E**). siRNA-mediated silencing of ACE2 has been shown to reduce SARS-CoV-2 pseudovirus entry in hACE2-expressing cells *in vitro*, with receptor recovery occurring within several days of treatment cessation (Tarnow et al., 2014). RISC-loaded siCX3CR1/F (19 bp, 2′-F/U) inhibits RSV G-binding >90% in HEp-2 (EC_50_ 0.5 nM), with lipid nanoparticles (LNP, 50 nm) achieving 60-70% silencing in bronchiolar epithelia; steric-blocking ASOs (morpholino-PMO, 25 mers) prevent splicing/translation of ICAM-1, curtailing rhinovirus >85% without RNase H. Sequential dosing (day 1/4) sustains protection through peak viral shedding, synergizing with mAbs for 5-log reductions; escape is negligible due to host target conservation.

#### Tissue-specific delivery challenges

Tissue-specific delivery challenges for pulmonary ASOs/siRNAs center on mucus/glycocalyx barriers, mucociliary clearance (t1/2 <20 min), and alveolar macrophage phagocytosis, with naked PS-ASOs achieving only 1-5% epithelial uptake versus 30-50% via LNPs or hyperbranched polylysines (Lee et al., 2018). Nasal turbinate dosing favours siRNA nebulisation (MMAD 1-3 μm), while deep lung deposition requires advanced formulations. Combination knockdown of ACE2/TMPRSS2 achieves synergistic entry suppression in air-liquid interface models (DeVincenzo et al., 2010).

### Host receptor modulation: safety and selectivity

Host receptor modulation is a promising antiviral strategy because it reduces the likelihood of resistance driven by viral mutation, yet its utility depends on maintaining a careful balance between antiviral activity and host safety (Zumla et al., 2016; de Wilde et al., 2017). Since many host receptors and associated proteases are embedded in essential signaling networks, therapeutic intervention must be selective enough to disrupt viral exploitation without broadly impairing normal cellular function (De Clercq and Li, 2016; Zumla et al., 2016). This is particularly important in respiratory viral infections, where the same molecules that facilitate entry or replication may also contribute to tissue homeostasis and epithelial integrity (Heurich et al., 2014; Hoffmann et al., 2020). Accordingly, the value of host receptor targeting lies not only in its antiviral efficacy, but also in its capacity to preserve physiological functions while limiting infection (de Wilde et al., 2017).

#### Physiological functions of target receptors

A key consideration in host-directed antiviral design is that the receptors exploited by viruses often serve indispensable biological roles in the host (De Clercq and Li, 2016; Zumla et al., 2016). These molecules regulate signaling, tissue repair, immune communication, and barrier maintenance, meaning that indiscriminate inhibition may produce unintended toxicity (de Wilde et al., 2017). Therefore, successful receptor-targeted therapies require a detailed understanding of expression patterns, tissue distribution, and functional redundancy (Zumla et al., 2016) This is especially relevant for receptors expressed in multiple organs, where systemic inhibition could disrupt both local and systemic homeostasis (Zumla et al., 2016).

#### Physiological roles of ACE2 in cardiovascular and renal homeostasis

ACE2 is a particularly important example because it functions both as a viral entry factor and as a regulator of cardiovascular and renal physiology (Chappell, 2016; Santos et al., 2018). In the renin-angiotensin system, ACE2 contributes to homeostasis by counterbalancing angiotensin II-mediated effects, thereby supporting vascular function and limiting tissue injury. Because of this protective role, complete ACE2 blockade may be biologically undesirable and potentially harmful (Santos et al., 2018). For this reason, therapeutic strategies should aim to interfere with viral interaction or downstream pathogenic effects while preserving ACE2’s normal homeostatic function (Santos et al., 2018; Hoffmann et al., 2020).

#### TMPRSS2 in epithelial biology

TMPRSS2 is another relevant host factor because it facilitates viral spike priming while also participating in normal epithelial biology (Meyer et al., 2014; Hoffmann et al., 2020). Its expression in epithelial tissues suggests that it contributes to tissue-specific protease balance and may influence epithelial differentiation or barrier-related processes (Lee et al., 2018). These physiological roles raise important safety concerns for systemic inhibition, particularly if treatment is prolonged or nonselective (de Wilde et al., 2017; Hoffmann et al., 2020). Nevertheless, TMPRSS2 remains an attractive antiviral target because its blockade can reduce viral entry, especially when inhibition is transient, localized, or combined with other antiviral approaches (Hoffmann et al., 2020).

#### Strategies for balancing antiviral efficacy with host physiological safety

The central challenge in receptor-based host-directed therapy is achieving sufficient antiviral efficacy without compromising essential host functions (Zumla et al., 2016; de Wilde et al., 2017). This balance can be improved by partial inhibition, tissue-restricted delivery, short treatment windows, or combination regimens that reduce the need for complete target suppression (De Clercq and Li, 2016; Zumla et al., 2016). In this context, the ideal host-directed antiviral strategy is not one that eliminates receptor function entirely, but one that selectively disrupts viral exploitation while preserving host physiology (de Wilde et al., 2017). Such an approach offers a more sustainable therapeutic framework, particularly for pathogens that rely on conserved host factors (Zumla et al., 2016).

### Temporal and spatial targeting strategies

#### Prophylactic versus therapeutic administration windows

Temporal and spatial targeting strategies enhance the therapeutic index of host-directed therapies (HDTs) by aligning drug exposure with the kinetics and localization of viral infection (Zumla et al., 2016; de Wilde et al., 2017). Respiratory viruses exhibit distinct temporal patterns of replication and spatial tropism within the airway epithelium, meaning that optimal HDT administration must synchronize with peak viral burden while prioritizing affected tissues. These approaches minimize off-target effects and improve efficacy compared to continuous systemic dosing, particularly for inhibitors of host entry factors like ACE2 or TMPRSS2 (Hoffmann et al., 2020). By exploiting the predictable progression of respiratory infections, temporal-spatial targeting transforms HDTs from broad-spectrum interventions into precision antivirals.

Prophylactic administration of HDTs offers distinct advantages over therapeutic use, particularly during high-risk exposure periods such as pandemics or seasonal surges (Dyall et al., 2017; Chan et al., 2020). Preemptive dosing can establish protective drug levels in the respiratory tract before viral challenge, blocking entry factors at the initial infection bottleneck where antiviral potency is greatest. In contrast, therapeutic administration requires higher doses to overcome established infection, increasing toxicity risks for host targets with physiological roles (de Wilde et al., 2017). Clinical evidence from SARS-CoV-2 prophylaxis trials supports short-term HDT use as a viable strategy for vulnerable populations, provided exposure risk is well-defined (Hoffmann et al., 2020).

#### Inhaled and intranasal delivery for respiratory tract selectivity

Inhaled and intranasal delivery routes achieve superior respiratory tract selectivity compared to systemic administration, concentrating HDTs at the primary site of viral replication (Lambert et al., 2005; Chan et al., 2020). Aerosolized formulations of TMPRSS2 inhibitors like camostat or nafamostat demonstrate high lung deposition with minimal plasma exposure, preserving systemic homeostasis while blocking surface entry pathways (Hoffmann et al., 2020). Similarly, intranasal ACE2 modulators can saturate nasal epithelial receptors, the initial SARS-CoV-2 infection portal, without affecting cardiovascular or renal ACE2 functions (Santos et al., 2018). These localized delivery systems represent a major advance in HDT optimization for respiratory pathogens.

#### Minimizing systemic exposure through localized respiratory delivery

Minimizing systemic exposure remains critical for HDTs targeting ubiquitously expressed host factors, as even transient off-target inhibition can disrupt homeostasis (De Clercq and Li, 2016; Zumla et al., 2016). Pharmacokinetic strategies including lung-restricted prodrugs, mucoadhesive nanoparticles, and short half-life inhibitors reduce circulation of active drug while maintaining therapeutic airway concentrations (Lambert et al., 2005). For example, nebulized camostat achieves >100-fold higher lung-to-plasma ratios compared to intravenous dosing, correlating with efficacy against coronaviruses without coagulopathy risks associated with systemic serine protease inhibition (Hoffmann et al., 2020). These exposure-minimizing approaches expand the safety window of HDTs for both prophylaxis and acute treatment.

## Clinical development landscape for host receptor-directed antiviral agents

Clinical development of host receptor-directed therapies has accelerated since the COVID-19 pandemic. While no agent has achieved regulatory approval for an entry receptor-blocking indication in adults, several candidates have generated proof-of-concept data (**Table 1**) (Andersen et al., 2020; Hammitt et al., 2022). The emerging picture is of a field with strong mechanistic foundations but inconsistent clinical translation, where trial design, timing of intervention, and patient selection have proven as determinative of outcomes as target biology itself (Martinez, 2020; McKee et al., 2020).

**Table 1:** Clinical trials of host receptor-directed antiviral therapies for respiratory viral infections.

Agent	Host/viral target	Virus	Trial phase	Modality	Key clinical outcome	Delivery route	Evidence status
Nirsevimab	RSV F protein (prefusion)	RSV	Phase III (MELODY trial)	Anti-receptor monoclonal Ab	75% reduction in RSV-associated hospitalization vs. placebo	IM injection	☑ Confirmed, licensed 2023
Clesrovimab	RSV F protein (prefusion, site IV)	RSV	Phase IIb/III (CLEVER, SMART)	Anti-receptor monoclonal Ab	~84% reduction in RSV hospitalization; ~61% reduction in medically attended LRTI vs. placebo	IM injection	Confirmed, licensed 2025
Palivizumab	RSV F protein	RSV	Licensed (since 1998)	Anti-receptor monoclonal Ab	~55% reduction in RSV hospitalization in high-risk premature infants	IM injection	☑ Confirmed, standard of care
Convalescent plasma	ACE2/spike (polyclonal)	SARS-CoV-2	Phase III (RECOVERY, n>10,000)	Polyclonal antibody preparation	No significant reduction in 28-day mortality or disease progression	IV infusion	☑ Confirmed null result
ALN-RSV01	RSV N gene (siRNA)	RSV	Phase II	siRNA (viral gene target)	2-log reduction in viral RNA in lung transplant patients; acceptable safety	Intranasal	☑ Confirmed
DAS181	Sialic acid receptors (sialidase)	Influenza/RSV	Phase II	Glycan competitor (receptor cleavage)	1.5-log reduction in viral shedding; no resistance emergence observed	Inhaled (DPI)	☑ Confirmed
Camostat mesylate	TMPRSS2	SARS-CoV-2/Influenza	Phase II	Small molecule inhibitor	0.5–1 log reduction in viral load; results inconsistent across RCTs	Oral	⚠ Partial, requires confirmation
Nafamostat	TMPRSS2/Furin	SARS-CoV-2	Phase II	Small molecule inhibitor	Improved PaO_2_/FiO_2_ in severe cases; no large powered RCT data	IV infusion	⚠ Partial, limited trial size
Soluble hrsACE2	ACE2 (viral decoy trap)	SARS-CoV-2	Phase II (case series)	Soluble receptor decoy	Viral load reduction reported in small case series; Phase II RCT pending	IV infusion	⚠ Case series only
Meplazumab	CD147 (Basigin)	SARS-CoV-2	Phase II RCT (n=44)	Anti-receptor monoclonal Ab	Reduced viral load and CRP; 87% discharge by day 10; not independently replicated	IV infusion	⚠ Single small trial
Bemcentinib	AXL receptor tyrosine kinase	SARS-CoV-2	Phase II (ongoing)	Kinase inhibitor (repurposed oncology)	Antiviral activity confirmed *in vitro*; robust clinical efficacy data lacking	Oral	⚠ Early-stage evidence

hrsACE2, human recombinant soluble ACE2; DPI, dry powder inhaler; VL, viral load; RCT, randomized controlled trial; mAb, monoclonal antibody; PaO_2_/FiO_2_, ratio of arterial oxygen partial pressure to fractional inspired oxygen.

☑ Green category: Confirmed evidence from adequately powered randomized trials or licensed regulatory approval.

⚠ Yellow category: Limited, early-stage, contested, or unreplicated evidence, requires confirmation in larger trials before clinical adoption.

Hammitt et al., 2022 (nirsevimab) (Hammitt et al., 2022); IMpact, 2014 (Pediatrics, 2014); RECOVERY Collaborative Group, 2021 (convalescent plasma); Yamaya et al., 2015 (camostat) (Yamaya et al., 2015); Zoufaly et al., 2020 (hrsACE2) (Zoufaly et al., 2020); Bian et al., 2020 (meplazumab) (Bian et al., 2020); DeVincenzo et al., 2010 (ALN-RSV01) (DeVincenzo et al., 2010); Yamamoto et al., 2020 (nafamostat) (Yamamoto et al., 2020).

### TMPRSS2 inhibitors

Camostat mesylate and nafamostat are the most clinically advanced small-molecule TMPRSS2 inhibitors. Camostat, a licensed serine protease inhibitor originally used in Japan for pancreatitis and esophageal reflux, demonstrated potent TMPRSS2 inhibition and SARS-CoV-2 entry blockade *in vitro* (Hoffmann et al., 2020). However, Phase II randomized controlled trials in ambulatory COVID-19 patients produced modest and inconsistent results, with some studies reporting 0.5–1 log reductions in viral load and others showing no significant effect on clinical progression (Meyer et al., 2014). Nafamostat, which has broader serine protease inhibition activity, showed improved oxygenation in small cohorts of severely ill COVID-19 patients receiving IV infusion, but large powered trials confirming hospitalization or mortality benefit are lacking (Yamamoto et al., 2020). Both agents are hampered by short half-lives and limited respiratory epithelium penetration when delivered systemically; inhaled formulations are under preclinical development as a more rational delivery strategy (Yamaya et al., 2015).

### Soluble ACE2 decoys

Recombinant human soluble ACE2 (hrsACE2) capitalizes on the high-affinity ACE2-spike interaction to intercept virions, with compassionate-use data showing viral load reduction (Zoufaly et al., 2020). Engineered dimeric ACE2-Fc constructs with substantially enhanced spike-binding affinity have demonstrated potent *in vitro* neutralization across SARS-CoV-2 variants, including Omicron sublineages, and represent the most promising next-generation iteration of this approach; however, adequately powered Phase II RCTs remain pending (Chan et al., 2020; Monteil et al., 2020). The primary safety concern, disruption of the renin-angiotensin system, has not proven a major clinical obstacle in short-course administration (Zoufaly et al., 2020).

### Anti-CD147 antibody (Meplazumab)

Meplazumab, a humanized anti-CD147 monoclonal antibody, was evaluated in a small open-label randomized trial (n=44) in hospitalized COVID-19 patients, with the treatment group showing reduced viral load and C-reactive protein with 87% discharge by day 10 (Bian et al., 2020). While promising, this trial was limited by its small size, open-label design, and the unresolved mechanistic question of whether CD147 functions as a bona fide entry receptor or primarily as an inflammatory mediator. Independent replication in larger, blinded trials is required before meplazumab can be considered validated as a receptor-directed antiviral agent (Shilts et al., 2021).

### RSV-directed receptor-blocking mAbs

The anti-RSV F protein mAbs and nirsevimab remain the most clinically advanced receptor-targeting agents in respiratory virology, as detailed in the RSV section above (Pediatrics, 2014). Nirsevimab, a next-generation long-acting mAb targeting the prefusion RSV F protein with extended half-life via YTE modifications, demonstrated 75% reduction in RSV-associated hospitalization versus placebo in the Phase III MELODY trial in healthy late-preterm and term infants, and received regulatory approval in multiple jurisdictions in 2023 (Hammitt et al., 2022). The field advanced further in 2025 with the U.S. FDA approval of a second long-acting anti-F monoclonal antibody, clesrovimab, administered as a single fixed 105-mg dose regardless of weight. In the CLEVER and SMART trials, it reduced RSV-associated hospitalizations by approximately 84% and medically attended lower respiratory tract infections by approximately 61% over a five-month season, and the Advisory Committee on Immunization Practices subsequently recommended it as an alternative to nirsevimab (Asturias et al., 2025). The rapid succession of two effective anti-F antibodies, together with real-world data showing roughly halved infant RSV hospitalization after their introduction, provides the clearest contemporary proof of concept that targeting a conserved viral-entry interface can yield durable, variant-resistant clinical protection, the strategic principle this review argues should now be extended to host-side entry determinants.

### Convalescent plasma

The RECOVERY trial, the largest randomized evaluation of convalescent plasma in COVID-19 (n>10,000 hospitalized patients), found no significant reduction in 28-day mortality or disease progression in the treatment arm compared to standard of care (Abani et al., 2021). Smaller earlier studies had suggested potential benefit in high-titer, early administration, but the overall evidence does not support convalescent plasma as a standard-of-care treatment for hospitalized COVID-19 (Joyner et al., 2024). Its limitations, lot-to-lot variability in neutralizing antibody titer, logistical complexity, and the difficulty of ensuring sufficient anti-receptor antibody fractions, highlight fundamental challenges of polyclonal preparations. The failure likely reflects several factors: by hospitalisation, viral replication has typically peaked and disease is driven by dysregulated immunity rather than ongoing entry; polyclonal antibody titres vary widely; and intravenous administration delivers inadequate concentrations to the respiratory mucosa. These lessons underscore the importance of early intervention, standardised potency metrics, and inhaled delivery for HDTs.

### siRNA and oligonucleotide approaches

ALN-RSV01, a siRNA targeting the RSV nucleocapsid (N gene) mRNA, demonstrated a 2-log reduction in viral RNA in lung transplant patients with established RSV infection in Phase II trials, with an acceptable safety profile (DeVincenzo et al., 2010). ALN-RSV01 targets a viral gene rather than a host receptor, but its development established key precedents for inhaled siRNA delivery to the respiratory epithelium that are directly relevant to host-receptor-silencing strategies currently under preclinical development. Host-receptor-targeting siRNA formulations, particularly ACE2 and TMPRSS2 silencers, have not yet entered clinical trials.

### DAS181 (receptor sialidase)

DAS181, a fusion protein consisting of a sialidase catalytic domain linked to an airway-anchoring domain, cleaves sialic acid receptors from the airway epithelial surface to prevent influenza HA binding. Phase II clinical trials demonstrated 1.5-log reductions in influenza shedding without emergence of resistance, with an inhaled delivery format that achieved high airway concentrations (Belser et al., 2013). Its mechanism, temporarily rendering the host cell surface non-permissive to influenza attachment, exemplifies the receptor-targeted strategy at its most direct. DAS181 remains under development for influenza and parainfluenza in immunocompromised patients.

### Combination therapies

#### Host-directed plus virus-directed approaches

The pharmacological rationale for combining host-directed entry inhibitors with direct-acting antivirals follows the principle established by HAART for HIV: simultaneous targeting of mechanistically independent steps in the viral lifecycle (extracellular entry blockade plus intracellular replication inhibition) raises the barrier to resistance beyond that achievable by either drug class alone, while potentially enabling dose reduction and improved therapeutic indices.

Preclinical synergy data support this rationale: camostat-remdesivir and sACE2-molnupiravir combinations show synergistic effects in cell-based models (Martinez, 2020; Ou et al., 2020). Nafamostat, a broader serine protease inhibitor with higher TMPRSS2 affinity than camostat, similarly showed additive-to-synergistic effects when combined with molnupiravir in primary human airway epithelial cell cultures, reducing infectious virus output by approximately 3 logs at clinically relevant concentrations (Andersen et al., 2020; Hoffmann et al., 2020). For MERS-CoV, the combination of soluble DPP4 decoy (sDPP4-Fc) with a DPP4 receptor-blocking monoclonal antibody has been evaluated in pseudovirus neutralization assays, demonstrating that the two agents targeting the same receptor through distinct epitopes achieve additive neutralization without apparent steric interference, providing a model for dual receptor-blockade strategies (Lu et al., 2013; Wang et al., 2013).

Key pharmacological principles for HDT-DAA combinations include: timing alignment (HDTs blocking entry are most effective within 24–48 hours of infection, whereas DAAs retain utility through 5 days of viral shedding); pharmacokinetic compatibility (camostat’s short half-life necessitates reformulation, and remdesivir’s IV-only route limits outpatient use); and synergy assessment through well-designed factorial studies (Hoffmann et al., 2020). Third, toxicity profiling for combinations targeting both a host receptor (with potential cardiovascular or metabolic consequences) and a viral enzyme (with potential hepatic or renal effects) requires dedicated safety evaluation beyond monotherapy data, particularly in the elderly and immunocompromised populations most likely to benefit.

The most immediate opportunity is pairing inhaled TMPRSS2 inhibitors with oral DAAs for outpatient treatment of COVID-19 and influenza.

## Pandemic preparedness

### Pre-positioned broad-spectrum strategies for emerging viral threats

The COVID-19 pandemic exposed the catastrophic cost of the gap between pathogen emergence and availability of specific countermeasures. This preparedness gap arises because virus-directed countermeasures require identification of the emerging pathogen’s specific genomic sequence or protein structure (Zumla et al., 2016; Kaufmann et al., 2017). This is the central pharmacological value of host receptor-directed approaches in pandemic preparedness: their efficacy is anchored in the biology of the host cell, not the virus, and is therefore expected to be independent of viral sequence variation within defined receptor-usage classes (Bekerman and Einav, 2015). This platform approach, targeting conserved host dependencies rather than strain-specific viral proteins, positions HDTs as first-line pandemic countermeasures deployable within weeks of pathogen identification (**Figure 2**).

The mechanistic basis for broad-spectrum potential rests on the convergence of phylogenetically distant virus families on a constrained set of host entry dependencies (Kaufmann et al., 2017). ACE2 is exploited by all sarbecoviruses; DPP4 by MERS-CoV and potentially other betacoronaviruses; sialic acid receptors by influenza and parainfluenza; and TMPRSS2 spans both families as a shared priming protease (Hoffmann et al., 2020). An HDT agent validated against one member of a receptor-sharing group therefore carries a biologically justified presumption of activity against newly emerged members of the same group, providing a rational basis for pre-positioning stockpiles of such agents before the next pandemic pathogen is identified (Zumla et al., 2016).

Three HDT categories are immediately actionable for pandemic preparedness: TMPRSS2 inhibitors (inhaled camostat/nafamostat), which retain activity against any coronavirus or influenza strain requiring TMPRSS2-mediated priming (Yamaya et al., 2015; Hoffmann et al., 2020). Second, soluble ACE2 decoys and anti-ACE2 antibodies provide a receptor trap strategy active against all sarbecoviruses that have co-evolved with human or bat ACE2, including undiscovered lineages with pandemic potential, since the host receptor sequence is fixed while the viral RBD continues to evolve (Chan et al., 2020; Monteil et al., 2020). Third, IgY polyclonal antibody preparations, produced by hyperimmunizing hens against conserved spike or fusion protein epitopes, offer a rapidly scalable, low-cost, mucosal-delivery platform that can be reformulated within 6–8 weeks of antigen availability, compared to the 12–18 months required for conventional monoclonal antibody manufacturing scale-up (Nguyen et al., 2010; El-Kafrawy et al., 2021). The broad epitope coverage of polyclonal IgY preparations further reduces the risk of resistance emergence through single viral escape mutations, a critical advantage in the early phase of an outbreak when viral diversity is rapidly expanding (El-Kafrawy et al., 2021).

#### Accelerating development timelines through platform approaches

The CEPI 100-Days Mission aims to compress vaccine development to 100 days, but even this ambitious timeline leaves a critical window during which HDTs could provide the only available pharmacological intervention (Kaufmann et al., 2017). This means that a validated TMPRSS2 inhibitor, an ACE2 decoy, or a broadly active IgY preparation could in principle enter emergency use evaluation within 30–60 days of a novel respiratory coronavirus emergence, a timeline far shorter than any pathogen-specific vaccine or mAb program (Zumla et al., 2016).

Realizing this potential requires investment in preparedness infrastructure: clinical-grade HDT stockpiles at regional distribution nodes, with stability data supporting adequate shelf-life for inhaled formulations and lyophilized preparations (Zumla et al., 2016). Second, adaptive platform trial infrastructure must be established in advance of the next pandemic, with pre-approved master protocols, pre-negotiated institutional agreements, and pre-positioned randomization systems capable of evaluating HDT candidates against novel pathogens within weeks of outbreak declaration, rather than months (Group, 2021). The RECOVERY and ACTIV platforms demonstrated that adaptive platform trials can evaluate multiple agents rapidly, a framework that should be extended to HDTs (Zumla et al., 2016; Kaufmann et al., 2017).

## Challenges and limitations

### On-target safety risks arising from essential host receptor functions

Host-directed therapies (HDTs) face significant challenges stemming from the essential physiological roles of their molecular targets, which complicates achieving antiviral efficacy without toxicity (Zumla et al., 2016; Martinez, 2020). While host factors offer resistance-proof targets, their ubiquitous expression across tissues creates narrow therapeutic windows that demand precise control of exposure, duration, and localization (de Wilde et al., 2017). Clinical translation has revealed modest monotherapy efficacy and heterogeneous patient responses, underscoring the need for combination strategies and refined patient stratification (Hoffmann et al., 2020). These limitations highlight that HDT success depends not just on target validation but on overcoming host biology constraints through innovative delivery and dosing paradigms.

The fundamental challenge of HDTs lies in targeting receptors and proteases that serve indispensable homeostatic functions beyond viral entry (Meyer et al., 2014; Santos et al., 2018). ACE2 maintains cardiovascular and renal balance through angiotensin-(1-7) production, while TMPRSS2 contributes to epithelial differentiation and prostate function, meaning complete inhibition risks disrupting normal physiology (Chappell, 2016). Even short-term blockade can trigger compensatory pathways or unmask functional redundancy, particularly in multi-organ systems where receptor expression overlaps with critical signaling networks (De Clercq and Li, 2016). This inherent conflict between antiviral need and physiological preservation defines the core limitation of receptor-based HDTs.

### Cardiovascular, renal, and metabolic safety considerations

ACE2 inhibition raises specific cardiovascular and renal safety concerns because it disrupts the renin-angiotensin system’s protective axis, potentially exacerbating hypertension, fibrosis, or acute kidney injury (Chappell, 2016; Santos et al., 2018). TMPRSS2 suppression carries reproductive toxicity risks due to its high prostate expression and role in androgen signaling, while broader protease inhibition may affect coagulation cascades (Meyer et al., 2014; Hoffmann et al., 2020). Clinical trials of camostat and nafamostat reported transient liver enzyme elevations and mild coagulopathy, highlighting the need for cardiac, renal, and hormonal monitoring during HDT administration (Group, 2021). These organ-specific risks necessitate careful patient selection and exposure minimization strategies.

### Gaps in long-term safety evidence for host receptor blockade

Long-term safety remains the most significant data gap for HDTs, as most candidates derive from short-term use in non-infectious indications without chronic host factor suppression data (Martinez, 2020; Sukhatme et al., 2021). Prolonged ACE2 or TMPRSS2 inhibition could alter tissue remodeling, immune surveillance, or endocrine balance in ways not captured by acute viral infection models (de Wilde et al., 2017). Post-marketing surveillance from repurposed drugs suggests acceptable tolerability for 7–14 day courses, but pandemic scenarios requiring extended prophylaxis create uncertainty about cumulative effects (Zumla et al., 2016). Addressing this gap requires longitudinal observational studies and chronic dosing models beyond current antiviral trial designs.

### Pharmacological challenges

Pharmacological optimization poses substantial hurdles for HDTs targeting membrane-bound receptors in polarized respiratory epithelium (Lambert et al., 2005; Hoffmann et al., 2020). TMPRSS2 localizes to the apical surface of airway cells, creating accessibility barriers for systemically delivered inhibitors, while endosomal cathepsins require lysosomal penetration inaccessible to many small molecules (Ou et al., 2021). Achieving sufficient local concentrations without systemic spillover demands advanced delivery systems, as plasma exposure correlates with toxicity while airway levels determine efficacy (Chan et al., 2020). These tissue-specific pharmacokinetic challenges distinguish HDTs from conventional antivirals targeting cytosolic viral enzymes.

#### Receptor accessibility and drug penetration in respiratory epithelium

Receptor accessibility represents a primary pharmacological barrier because TMPRSS2 and ACE2 localize to the airway luminal surface, separated from systemic circulation by tight junctions and mucus barriers (Heurich et al., 2014; Hoffmann et al., 2020). Inhaled delivery improves apical access but faces mucociliary clearance and variable lung deposition, while oral agents struggle to achieve therapeutic airway concentrations without gut metabolism or hepatic first-pass effects (Lambert et al., 2005). Human airway organoid studies confirm that only ~10-20% of systemically delivered protease inhibitors reach functional levels at the apical membrane, underscoring the need for mucoadhesive or nanoparticle-enhanced formulations (Ou et al., 2021).

#### Optimal dosing regimens

Optimal HDT dosing remains poorly defined due to nonlinear pharmacokinetics and host adaptation during viral clearance (de Wilde et al., 2017; Martinez, 2020). Early intensive dosing blocks viral entry most effectively, but prolonged exposure increases toxicity risk, while delayed administration reduces efficacy against established infection (Hoffmann et al., 2020). Pharmacodynamic models suggest 3–5 day “pulse” regimens achieve >90% target inhibition during peak replication with minimal cumulative exposure, though individual variability in receptor expression requires personalized adjustments (Sukhatme et al., 2021). Adaptive dosing guided by viral load or biomarker response represents the optimal paradigm pending prospective validation.

#### Targeted drug delivery to primary sites of respiratory viral replication

Efficient drug delivery to infection sites constitutes a critical bottleneck for HDT efficacy in the distal respiratory tract (Lambert et al., 2005; Chan et al., 2020). While upper airway infections respond to intranasal administration, lower respiratory involvement requires aerosol formulations achieving deep lung deposition without macrophage clearance or airway irritation (Hoffmann et al., 2020). Nanoparticle carriers, mucoadhesive polymers, and dry powder inhalers enhance retention and penetration through mucus barriers, but scale-up manufacturing and cold chain requirements limit pandemic deployment (Group, 2021). Device-agnostic liquid formulations compatible with standard hospital nebulizers offer the most practical solution for emergency use.

### Regulatory and development pathways

HDTs challenge conventional regulatory frameworks designed for pathogen-specific antivirals, requiring novel approval pathways that balance host safety against outbreak urgency (Martinez, 2020; McKee et al., 2020). Repurposed drugs leverage existing safety data for accelerated EUA, but *de novo* HDTs face prolonged toxicology requirements due to host target concerns (Zumla et al., 2016). Platform trial designs like ACTIV and PRINCIPLE enable simultaneous testing across multiple HDT candidates, compressing development timelines while generating comparative efficacy data (Sukhatme et al., 2021). Conditional approval based on viral clearance and biomarker endpoints, rather than mortality alone, better aligns with HDT mechanism and early intervention paradigm.

#### Navigating novel regulatory approval frameworks for host-directed antivirals

The novelty of HDT mechanisms necessitates evolution in regulatory approval frameworks beyond traditional antiviral endpoints (Andersen et al., 2020; McKee et al., 2020). Time-to-viral clearance and reduction in viral burden serve as more proximal efficacy measures than hospitalization rates, particularly for ambulatory early treatment (Martinez, 2020). Host biomarker panels tracking receptor occupancy, inflammation resolution, and tissue protection provide mechanistic validation absent in virologic endpoints alone (de Wilde et al., 2017). Adaptive platform trials with seamless Phase II-III progression and real-world evidence incorporation address both speed and rigor requirements unique to host-targeted pandemic countermeasures (Zumla et al., 2016).

#### Clinical trial endpoint selection for host-directed antiviral therapies

Appropriate endpoints for HDTs must reflect their early intervention mechanism rather than late-stage disease progression metrics (Group, 2021; Sukhatme et al., 2021). Primary endpoints should prioritize time to viral undetectability, area-under-curve viral load reduction, and symptom resolution within 7–14 days, as HDTs exert greatest impact during the pre-symptomatic replication phase (Hoffmann et al., 2020). Secondary host response endpoints including cytokine normalization, tissue oxygenation surrogate markers, and receptor occupancy via plasma proteomics provide mechanistic corroboration (Martinez, 2020). Composite endpoints combining virologic, clinical, and host biomarker outcomes offer the most comprehensive assessment of HDT impact across the infection cascade.

## Conclusions and future directions

### Synthesis of the host receptor paradigm

The evidence reviewed here supports host receptor-directed therapy as a scientifically promising and mechanistically distinct approach in respiratory antiviral development. Across the full spectrum of respiratory viral pathogens, from seasonal influenza and RSV to pandemic-potential coronaviruses, the fundamental dependence of viruses on conserved host cell-surface molecules for attachment, proteolytic priming, and membrane fusion creates a set of therapeutic vulnerabilities that are structurally stable, mechanistically well-defined, and potentially resistant to viral mutational escape (Bekerman and Einav, 2015; Kaufmann et al., 2017). The diversity of available modalities creates a toolkit for matching intervention to clinical scenario (Zumla et al., 2016; Dyall et al., 2017).

The central advantage of this paradigm, that host targets do not co-evolve with the virus, has been supported by *in vitro* and preclinical evidence. TMPRSS2 inhibitors retained activity across SARS-CoV-2 variants from ancestral Wuhan through Omicron sublineages in cell-based assays despite profound changes in spike protein structure, although clinical translation of this preserved activity has been inconsistent (Hoffmann et al., 2020). Soluble ACE2 decoys neutralized pseudoviruses bearing spike proteins from multiple sarbecovirus lineages, demonstrating cross-species breadth that no spike-directed monoclonal antibody can match (Chan et al., 2020; Monteil et al., 2020). Anti-sialic acid glycan inhibitors retained activity against oseltamivir-resistant H1N1 and avian H5N1 strains, underscoring how decoupling antiviral activity from viral surface protein variability fundamentally changes the durability of the therapeutic effect (Matrosovich et al., 2009; Bhatia et al., 2016). These examples suggest that host-directed strategies may complement virus-directed therapies by providing an independent pharmacological layer targeting the entry step of infection. Whether this translates into clinically meaningful benefit in powered trials remains the central unanswered question in the field.

It is therefore useful to compare host-directed and direct-acting antivirals explicitly rather than to frame them as competitors. Regarding clinical efficacy, direct-acting agents currently hold a decisive advantage: oral 3CLpro and polymerase inhibitors have demonstrated outpatient benefit in large randomized trials, whereas no host-receptor-directed antiviral has not yet shown comparable efficacy in an adequately powered endpoint trial. On durability and breadth, the balance is reversed in principle: host targets do not co-evolve with the virus, so a receptor-directed agent should resist the antigenic and protease-site escape that erodes antibody and, increasingly, DAA activity, though this advantage remains largely theoretical pending clinical confirmation. On safety, direct-acting antivirals benefit from targeting non-self viral enzymes, while host-directed agents carry intrinsic on-target risk from interfering with the physiological functions of ACE2, DPP4, and TMPRSS2, making therapeutic-window and delivery considerations more demanding. On cost and implementation, small-molecule DAAs are generally cheaper and simpler to distribute than biologics, although low-cost platforms such as IgY and the strain-agnostic nature of some host-directed agents could offset this in outbreak and resource-limited settings. The distinct novelty of the present review is to draw these axes together across multiple respiratory virus families within a single pharmacological framework: rather than asking whether host-directed therapy can replace direct-acting antivirals, we argue that its realistic near-term value lies in defined complementary niches (prophylaxis, early treatment, combination regimens, and pandemic bridging). The receptor-by-receptor and modality-by-modality evidence assembled here is intended to make that positioning concrete.

### Critical unresolved challenges

A second major challenge is the delivery of nucleic acid-based receptor-silencing strategies, siRNA and antisense oligonucleotides targeting ACE2, TMPRSS2, or sialic acid biosynthetic enzymes, to the relevant respiratory epithelial cells *in vivo*. While lipid nanoparticle (LNP) and GalNAc conjugate platforms have transformed the hepatic delivery of oligonucleotide therapeutics, efficient, durable, and non-inflammatory delivery to ciliated airway epithelium remains technically demanding, and the transient nature of receptor silencing may require dosing regimens incompatible with prophylactic use in pandemic scenarios (Kulkarni et al., 2018; Setten et al., 2019). Progress in inhaled LNP formulations and mucus-penetrating nanoparticle systems offers a plausible path forward, but rigorous *in vivo* validation in large-animal respiratory models is needed before clinical translation.

### Priority areas for future research

Building on the synthesis above, the following research priorities are identified as most likely to advance the field toward clinical impact:

1. Localized delivery platforms. Advancing inhalable formulations (DPIs, nebulized solutions) for direct respiratory mucosal targeting of HDTs. Pharmacokinetic and pharmacodynamic optimization studies in non-human primate respiratory tract models are needed (Yamaya et al., 2015; Monteil et al., 2020; El-Kafrawy et al., 2022).
2. Combination HDT-DAA regimens. Prioritizing rational combinations, particularly inhaled TMPRSS2 inhibitors with oral DAAs, through factorial trial designs (Zumla et al., 2016; Dyall et al., 2017).
3. Pan-receptor platforms. Developing agents that simultaneously target multiple entry receptors for maximal breadth and resistance barriers (Bekerman and Einav, 2015; Dyall et al., 2017).
4. Rigorous *in vivo* safety studies for receptor modulation examining cardiovascular, renal, and immunological consequences of sustained receptor blockade (Drucker, 2007; Haga et al., 2008).
5. Standardized preclinical frameworks. Establishing consensus models, potency assays, and evaluation criteria for HDT candidates. *in vivo* model selection, and pharmacokinetic characterization before clinical translation is pursued (Bekerman and Einav, 2015).
6. IgY and alternative antibody platforms. Avian IgY antibodies represent a platform for mucosal host-receptor blockade that combines low production cost, scalability, epitope diversity, and a favorable safety profile in preclinical models. However, it should be noted that the clinical evidence base for IgY in respiratory viral infections remains at an early stage, with no completed Phase II RCTs, and the degree of attention given to this platform in the current review reflects the authors’ assessment of its future potential rather than established clinical validation (Nguyen et al., 2010; Rahman et al., 2013; El-Kafrawy et al., 2022). Future work should focus on formulation stability for intranasal and inhaled delivery, breadth of neutralization across viral variants, and clinical evaluation in randomized controlled trials in high-risk populations.

### Future perspectives: a three- to five-year outlook

Translating the priorities above into measurable progress will require concrete near-term milestones. Within the next three to five years, we suggest that the field should prioritize, in order of tractability: (i) at least one adequately powered, placebo-controlled trial of an inhaled TMPRSS2 inhibitor in early outpatient influenza or COVID-19, designed with virological and clinical co-primary endpoints and with pharmacokinetic confirmation of airway target engagement, since the protease axis is the most clinically mature and its previous failures are plausibly attributable to delivery and timing rather than to the target itself; (ii) first-in-human and Phase II evaluation of an engineered high-affinity soluble ACE2 decoy delivered by the inhaled or intranasal route, leveraging the variant-agnostic binding demonstrated preclinically; (iii) a factorial or adaptive-platform trial formally testing a host-directed plus direct-acting antiviral combination, to quantify any additive or synergistic benefit and to characterize drug–drug interactions and resistance suppression directly rather than by inference; (iv) prospective evaluation of a pan-DPP4-blocking antibody as a pre-pandemic MERS-CoV countermeasure, advanced through the regulatory pathways available for emerging-pathogen medical countermeasures; and (v) controlled clinical evaluation of a mucosally delivered polyclonal (including IgY) preparation in a high-risk or outbreak setting, paired with standardized potency and stability metrics. Realizing these milestones will depend on regulatory adaptation, agreed surrogate and clinical endpoints for host-directed antivirals, and frameworks that allow stockpiling and rapid deployment of broad-spectrum agents before a specific pathogen is identified. Equally important is explicit attention to cost-effectiveness and deliverability relative to established direct-acting antivirals, so that agents reaching approval are also feasible to deploy in the resource-limited settings that bear much of the global respiratory-virus burden.

### Limitations of this review

Several limitations of this review should be acknowledged. First, the authors of this review have active research programs in IgY antibody development and MERS-CoV host-receptor targeting, which may influence the emphasis and framing of certain sections. We have endeavoured to present the evidence objectively, but readers should be aware of this potential source of bias. Second, this review is narrative rather than systematic, and the selection of included studies was based on the authors’ assessment of relevance rather than on pre-specified inclusion and exclusion criteria; important studies may therefore have been inadvertently omitted. Third, the rapid pace of publication in the host-directed therapy field means that some clinical trial results or mechanistic studies published after our literature search may not be captured. Fourth, for several therapeutic modalities discussed, the available evidence is predominantly preclinical, and the extrapolation from *in vitro* and animal model data to likely clinical efficacy involves substantial uncertainty that may not be fully conveyed in all sections. Finally, the review is weighted toward SARS-CoV-2 and MERS-CoV receptor biology, reflecting the concentration of recent research activity, and coverage of influenza and RSV host receptor targeting, while included, may be comparatively less comprehensive. Several further constraints apply to the evidence base itself rather than to our synthesis. Because we did not apply a formal risk-of-bias or quality-assessment instrument, the strength of individual studies was judged qualitatively; the field is also susceptible to publication bias, as positive preclinical and early-phase results are more likely to be reported than negative ones, which may inflate the apparent promise of some modalities. In addition, the great majority of the clinical and preclinical studies cited originate from high-income settings, and questions of cost, scalability, cold-chain stability, and deliverability in low- and middle-income countries, where much of the global respiratory-virus burden falls, remain underexplored; agents such as low-cost, heat-stable IgY preparations may be particularly relevant here but require dedicated evaluation. Finally, long-term safety data for sustained host-receptor modulation are largely lacking: most clinical exposures to date are short (often on the order of two weeks), so the chronic cardiovascular, renal, metabolic, and immunological consequences of prolonged or repeated receptor blockade, especially relevant for prophylactic use, cannot yet be assessed and constitute an important evidence gap.

### Concluding statement

Respiratory viruses will continue to emerge, evolve, and challenge public health preparedness systems for the foreseeable future. Strategies that target the host rather than the virus offer a complementary approach that is, on current evidence, theoretically more resistant to viral escape and potentially broader in spectrum. These properties have so far been demonstrated mainly *in vitro* and in animal models rather than in adequately powered clinical trials. Such strategies may nonetheless be well suited to the early phase of a pandemic before pathogen-specific countermeasures are available. However, the major scientific and translational challenges identified in this review, including on-target safety, delivery optimization, modest clinical efficacy data to date, and the need for standardized evaluation frameworks, must be addressed through focused investment and cross-disciplinary collaboration between virologists, structural biologists, pharmacologists, and clinical trialists. While host receptor targeting has generated promising preclinical and early clinical data, the priority now is rigorous, well-powered clinical translation that can determine whether the biological promise of this approach translates into meaningful clinical benefit under the evidentiary standards required for regulatory approval and public health deployment.
